# ADCY3: the pivotal gene in classical ketogenic diet for the treatment of epilepsy

**DOI:** 10.3389/fncel.2024.1305867

**Published:** 2024-05-22

**Authors:** Mingxing Lin, Jiayin Gong, Luyan Wu, Xin Lin, Yuying Zhang, Wanhui Lin, Huapin Huang, Chaofeng Zhu

**Affiliations:** ^1^Department of Pediatrics, Fujian Medical University Union Hospital, Fuzhou, China; ^2^Department of Neurology, Fujian Medical University Union Hospital, Fuzhou, China; ^3^Fujian Key Laboratory of Molecular Neurology, Fuzhou, China; ^4^Department of Geriatrics, Fujian Medical University Union Hospital, Fuzhou, China

**Keywords:** ketogenic diet, fatty acid metabolism, ADCY3, CAMP signaling pathway, epileptic epilepsy, neuronal inhibition

## Abstract

**Objective:**

Epilepsy is a common neurological disorder characterized by recurrent epilepsy episodes. As a non-pharmacological treatment, the ketogenic diet has been widely applied in treating epilepsy. However, the exact therapeutic mechanism of the ketogenic diet for epilepsy remains unclear. This study investigates the molecular mechanisms of the ketogenic diet in regulating fatty acid metabolism and activating the ADCY3-initiated cAMP signaling pathway to enhance neuronal inhibition and thereby treat epilepsy.

**Methods and results:**

Meta-analysis reveals that the ketogenic diet is superior to the conventional diet in treating epilepsy. Animal experiments demonstrate that the ketogenic diet is more effective than the conventional diet in treating epilepsy, with the best results achieved using the classic ketogenic diet. Transcriptome sequencing analysis identifies six essential genes, among which ADCY3 shows increased expression in the ketogenic diet. In vivo experiments confirm that the activation of the cAMP-PKA signaling pathway by ADCY3 enhances neuronal inhibition and improves epilepsy control.

**Conclusion:**

Clinical observations indicate that the ketogenic diet improves patient epilepsy episodes by regulating the ADCY3-initiated cAMP signaling pathway.

## 1 Introduction

Epilepsy remains a prevalent neurological disorder, manifesting in recurrent seizures that significantly impact the quality of life for many individuals (Beghi et al., 2015; Manford, 2017; Thijs et al., 2019; Levin et al., 2021). Although an array of anticonvulsants drugs are available, they fail to provide substantial relief for a significant subset of patients, either due to drug resistance or intolerable adverse effects (Klein et al., 2021). This prevailing issue underlines the pressing need for innovative and effective alternative treatments (Sorkpor and Ahn, 2021). One such promising non-pharmacological approach is the ketogenic diet, which has garnered empirical support for its potential therapeutic benefits in managing epilepsy (Sampaio, 2016; Paoli et al., 2020; Sourbron et al., 2020; Wells et al., 2020). Nonetheless, the intricate mechanisms underpinning its anticonvulsants effects remain partially obscured, necessitating further comprehensive exploration (Zhang et al., 2021a).

The ketogenic diet is fundamentally high in fats, moderate in proteins, and low in carbohydrates. It uniquely simulates a fasting state by inducing the production of β-hydroxybutyrate, a key metabolic product (Youm et al., 2015; Beam et al., 2021; Khoziainova et al., 2022). While the exact mechanisms of the anticonvulsants effects of the ketogenic diet are not fully understood, studies have shown that the diet can influence various metabolic pathways and signaling pathways in the brain, thereby reducing the frequency and severity of seizures (Zarnowska, 2020). Literature reports indicate that the ketogenic diet offers an effective alternative for children and adults with drug-resistant epilepsy, showing particular efficacy in certain epilepsy syndromes such as West syndrome, severe infantile spasms, myoclonic-astatic epilepsy, fever-related epilepsy syndromes, and drug-resistant idiopathic generalized or refractory epilepsy (Elia et al., 2017). Furthermore, in drug-resistant epilepsy, the ketogenic diet has demonstrated utility (Ułamek-Kozioł et al., 2019). Ketogenic diet-induced increase in circulating ketones may assist in the treatment of drug-resistant epileptic episodes (Rogawski et al., 2016). The production of ketone bodies in the liver as a result of the ketogenic diet helps control seizures through its anticonvulsant action (Cicek and Sanlier, 2023). When conventional anticonvulsants medications and anesthetics fail, the ketogenic diet presents a promising new adjunctive strategy for managing acute status epilepticus in the intensive care setting (McDonald and Cervenka, 2020). Research suggests that the ketogenic diet is high in fat and protein content but low in carbohydrates, with β-hydroxybutyrate being the primary ketone body produced during carbohydrate deficiency in the diet, believed to have neuroprotective effects (Polito et al., 2023). Additionally, studies on the ketogenic diet often consider β-hydroxybutyrate as a crucial marker (Lin et al., 2023; Martins et al., 2023; Poorshiri et al., 2023). Moreover, literature indicates that β-hydroxybutyrate is a byproduct of normal fatty acid oxidation metabolism. In animals, β-hydroxybutyrate serves not only as an intermediary metabolite but also as a significant regulatory molecule that can influence gene expression, lipid metabolism, neuronal function, and overall metabolic rate (Mierziak et al., 2021).

Fatty acid metabolism is a central feature of the ketogenic diet (Nagpal et al., 2019; Kraeuter et al., 2020). By enhancing fatty acid metabolism, the ketogenic diet can lead to the production of abundant β-hydroxybutyrate in the body (Koronowski et al., 2021; Basolo et al., 2022; Karagiannis et al., 2022). Recent studies have revealed a close relationship between fatty acid metabolism and epileptic seizures (Samanta, 2021). ADCY3 (Adenylate Cyclase 3), a crucial signaling molecule, is involved in regulating intracellular levels of cyclic adenosine monophosphate (cAMP) (Wu et al., 2016; Son et al., 2022; Gárriz et al., 2023). Research has detected miRNA in the cerebrospinal fluid of subjects, identifying potential target cells of ADCY3 including the choroid plexus, neurons, and microglial cells (Narayan et al., 2020). ADCY3 plays a significant role in neuronal excitability and is associated with severe depression (Yang et al., 2021). Furthermore, a study comparing two weight-loss diets has demonstrated that the ADCY3 gene is involved in regulating various metabolic processes, such as the development and function of adipose tissue, with specific regulatory effects depending on the nutrient intake levels (Goni et al., 2018). Therefore, we hypothesize that the ketogenic diet may regulate ADCY3 by promoting fatty acid metabolism, further activating the cAMP signaling pathway, influencing neuronal inhibition, and improving epileptic seizures.

To test this hypothesis, a comprehensive experimental design was implemented. Initially, a meta-analysis was conducted to evaluate the efficacy of ketogenic diet on epileptic seizures. Subsequently, animal experiments were carried out using an epilepsy mouse model to observe the effects of different types of ketogenic diets on seizure activity, assessing their anticonvulsants effects through indicators such as electroencephalograms. Furthermore, key genes and signaling pathways related to the ketogenic diet were identified through transcriptome sequencing and protein interaction analysis. Lastly, a mouse model overexpressing ADCY3 was constructed, followed by *in vivo* validation using a cAMP inhibitor, to elucidate the role of the ADCY3/cAMP signaling pathway in the ketogenic diet.

Our study results are expected to further elucidate the mechanistic role of ketogenic diet in treating epilepsy, providing a theoretical basis and new therapeutic strategies for its clinical application. Understanding the effects of ketogenic diet on fatty acid metabolism, ADCY3 activation of the cAMP signaling pathway, and regulatory mechanisms of neuronal inhibition holds significant importance for investigating the pathogenesis of epileptic seizures and treating related disorders.

## 2 Materials and methods

### 2.1 Meta-analysis

Searching computer databases such as PubMed, Cochrane Library, Embase, and Web of Science. The retrieval is set from the establishment of the database until April 2023. Search is performed using a combination of controlled vocabulary and free words. The retrieval terms primarily include: “Epilepsy” OR”Epilepsies,” “Seizure Disorder,” “Seizure Disorders,” “Awakening Epilepsy,” “Epilepsy, Awakening, “Epilepsy, Cryptogenic” OR”Cryptogenic Epilepsies” OR”Cryptogenic Epilepsy” OR”Epilepsies, Cryptogenic” OR”Aura” OR”Auras.”

The inclusion criteria for the literature meets the principles of evidence-based medicine PICOS (P-patient, I-intervention, C-comparison intervention, O-outcomes): (1) Patient population: patients diagnosed with epilepsy; (2) Intervention: ketogenic diet (KD); Comparison group: comparison with regular diet or different KDs; (3) Study design: clinical randomized controlled trials; (4) Outcome measures: frequency of epileptic epilepsy, including epilepsy reduction ≥50% or epilepsy reduction ≥90% or no epilepsy; (5) Included literature should include general information about the patients, adverse reactions, follow-up, and number of dropouts.

Exclusion criteria include (1) non-randomized controlled trials (RCTs); (2) studies that do not include relevant outcome measures and treatment methods; (3) incomplete literature data integrity; (4) non-case–control experiments; (5) duplicate publications; (6) guidelines, conference reports, systematic reviews or abstract articles; (7) animal experiments; (8) non-English literature.

The literature included in the study was independently analyzed by two researchers. Use Endnote software to identify relevant literature by reading the titles, abstracts, and full texts to determine if they meet the inclusion criteria. Disagreements are resolved through consensus to ensure the objectivity and integrity of the data.

According to the Jadad scale, the methodological quality of incorporating data is evaluated based on four aspects: random allocation method (appropriate, unclear, inappropriate), concealment of allocation scheme (appropriate, unclear, inappropriate), blinding method (appropriate, unclear, inappropriate), and follow-up records (present, absent). Scores 1 to 3 indicate low-quality research, while scores 4 to 7 represent high-quality research (Wells et al., 2020).

### 2.2 Generation of epileptic mouse model and sample acquisition

This experiment used healthy C57BL/6 male mice (weighing 20–30 g, 8–10 weeks old). All mice were purchased from the Experimental Animal Center of Guangdong Province and were housed in SPF-grade laboratories. The housing conditions met the standard requirements, including humidity (44–78%), temperature (17–20°C), lighting (12-h light–dark cycle), and access to food and water. The mice were acclimated to the experimental environment for 1 week. All mice were handled strictly with the ethical requirements for experimental animals and obtained approval from the Animal Ethics Committee for Experimentation.

The mice were randomly divided into six groups: a regular group of 10 mice (Control), a model group of 10 mice with epilepsy (Model), a group of 10 mice on a classical ketogenic diet (Model+KD), a group of mice on a medium-chain triglyceride ketogenic diet (Model+MCT), a group of mice on a modified Atkins diet (Model+MAD), and a group of mice on a low glycemic index ketogenic diet (Model+LGIT).

Proportions of nutrients in different types of ketogenic diets: Classic KD: fat content (80%); low carbohydrate content (5%); protein content (15%) (Neal et al., 2009). MCT: MCT composition contains two fatty acids, caprylic acid (C: 8.60%) and capric acid (C: 10.40%) (Hollis et al., 2018; Guimarães et al., 2019). MAD: carbohydrate content (7.6%), fat content (54.5%), protein (21.2%) (Gupta et al., 2021). LGIT: fat content (65%); low carbohydrate content (25%); protein content (10%) (Karimzadeh et al., 2014).

The KA (kainic acid) model is a classical model that simulates human temporal lobe epilepsy and is widely used in the study of refractory epilepsy drugs. Rodents can induce epileptic status after intraperitoneal injection of KA, which activates the limbic system (Grabenstatter et al., 2005).

Adult mice (20–30 g) were induced with a cell cytotoxic epilepsy model by intraperitoneal injection of KA (kainic acid) (55001ES10, Yeasen). Administer a low dose (5 mg/kg) every hour until mice exhibit a sustained epilepsy lasting longer than 3 h. After inducing epilepsy in each mouse, a subcutaneous injection of approximately 2.5 milliliters of lactated Ringer’s solution (approval number: National Medical Products Administration H20059425, Jiseng) was administered (Grabenstatter et al., 2005; Modebadze et al., 2016; Ramazi et al., 2022). Record each mouse’s epilepsy severity and convulsion duration according to the Racine Scale. In surviving mice, select mice that exhibit spontaneous and recurrent epilepsy within a few days and continue with subsequent experiments for several weeks. Inject an equivalent dose of normal saline into the control group. The standard experimental group and the epileptic mouse model group were fed a conventional diet, while the mice in different types of ketogenic diet groups were fed with different types of ketogenic feed (Wang et al., 2021) and purchased from the high-tech platform for experimental animal feed. We used a video monitoring method to continuously observe mice for 3 months and recorded information, including the frequency and duration of epileptic epilepsy (Modebadze et al., 2016; Wang et al., 2021).

To collect samples, excessive intraperitoneal injection of 0.5% pentobarbital sodium (controlled substance, contact customer service for purchase) or (P3761, Sigma) was used to euthanize the mice. Harvest the mouse brain hippocampal tissue and quickly wash the surface stains with physiological saline. Remove excess liquid and cut the tissue into specified sizes, collecting them in pre-chilled enzyme-free tubes. Place the tubes in liquid nitrogen and quickly freeze them at −80°C for storage after collecting all the samples (López-Rivera et al., 2023).

### 2.3 Generation of an animal model of overexpression of ADCY3 in epileptic mice

Divide the mice into four groups: oe-NC (overexpressing lentivirus negative control group), oe-ADCY3 (overexpressing lentivirus ADCY3 group), oe-ADCY3 + DMSO, and oe-ADCY3 + RMI12330A (cAMP inhibitor RMI 12330A) (Guellaen et al., 1977; Grupp et al., 1980; Griebel et al., 1991; Kim et al., 2019). Each group has 10 mice. The design customization service of RMI12330A is provided by Shanghai Yiji Bio-Technology Co., Ltd. Inducing epilepsy in mice using pilocarpine has been described earlier. After confirming the successful establishment of an epileptic mouse model, the ADCY3 overexpression was carried out using the lentiviral transduction method for 4 weeks. Subsequently, OE-ADCY3 + RMI12330A group mice were intraperitoneally injected with RMI 12330A (dissolved in DMSO at 15 mg/kg). The mice in the oe-ADCY3 + DMSO group were also injected with an equivalent dose of DMSO solution (Hong et al., 2013).

### 2.4 Lentivirus transfection

Epileptic mice overexpressing ADCY3 were constructed using a lentiviral transfection method. Initially, the pAcGFP plasmid vector was appropriately reconstructed by replacing the original CMV promoter with the SYN1 gene promoter. This promoter drives the expression of the SYN1 gene, producing Synapsin I protein, which exhibits specific expression within neurons, enabling the specific expression of ADCY3 in neuronal cells (Wang et al., 2018). Plasmid design, construction, and lentiviral packaging services were provided by Shanghai’s Biotech Engineering Company. Four hundred nanogram of pAcGFP-ADCY3 was transfected into HEK293 cells (CL-0005) using Lipofectamine 2000, followed by validation, amplification, and purification to obtain the packaged lentivirus. Mice were secured in appropriate restraint devices, with the injection needle positioned at the base of the tail to ensure entry into the tail vein. Subsequently, the prepared lentivirus was slowly injected into the tail vein of the mice (titer: 5 × 10^6^ TU/mL) once a week for a total of 4 weeks (Hong et al., 2013).

### 2.5 Ketogenic diet treatment for preparation of mouse models of epilepsy and injection of cAMP inhibitors

The methods for purchasing mice and establishing the epilepsy model were performed as described previously. Mice were randomly assigned into two groups, with 10 mice in each group: KD group and KD + RMI 12330A group. Following the successful establishment of the epilepsy model, the mice were fed a ketogenic diet for 3 months. On the 90th day, mice in the KD + RMI 12330A group received an intraperitoneal injection of RMI 12330A (a cAMP inhibitor, 15 mg/kg), after which relevant parameters were measured and observed (Guellaen et al., 1977; Grupp et al., 1980; Biondi et al., 1990; Griebel et al., 1991; Hong et al., 2013; Kim et al., 2019).

### 2.6 Behavioral observation of mice (Racine scoring)

Evaluation criteria: R1: The mildest epileptic epilepsy is manifested as bright, flashing, or unusual sensations in the field of vision, referred to as photosensitivity or visual abnormalities, or localized muscle spasms, also known as focal epilepsy. R2: Mild epileptic epilepsy, characterized by local muscle twitching or jerking, which can spread to adjacent muscles, are called clonic epilepsy. R3: Moderate seizure, manifested as a grand mal seizure, where the patient suddenly loses consciousness, experiences muscle convulsions or stiffness, and may have symptoms such as foaming at the mouth and urinary incontinence. R4: Severe epileptic epilepsy, manifested as grand mal epilepsy, sudden intense emotional reactions such as fear or anger, possibly accompanied by loss of consciousness, muscle convulsions or stiffness, foaming at the mouth, incontinence, rapid breathing, etc. R5: The most severe epileptic epilepsy is characterized by a status epilepticus, which involves a prolonged period of unconsciousness until the epilepsy episode ends and may result in severe brain damage (Van Erum et al., 2019).

### 2.7 EEG measurements in epileptic mice

Anesthesia was induced in mice by intraperitoneal injection of 0.5% sodium pentobarbital at 50 mg/kg. Bipolar, coated stainless steel electrodes were implanted in the right CA3 region, with a single reference electrode and ground electrode fixed above the mouse brain cortex and cerebellum. Twenty-four hours after the implantation surgery, we used Pinnacle’s Nervus EEG recording system to record a 12-h electroencephalogram (EEG). We define an epileptic epilepsy as a sustained high-amplitude rhythmic discharge (such as repeated spike waves, spike-and-wave complexes, and slow waves) lasting at least 5 s, with a frequency greater than 5 Hz and an amplitude greater than twice the baseline (Masino et al., 2011). Later, after 3 months of ketogenic treatment, the brainwave of the mice was recorded.

### 2.8 High-throughput transcriptome sequencing

Three hippocampal tissue samples were selected from the standard group, the epilepsy model group, and the classic ketogenic diet group, respectively. Each sample was extracted for at least 1 μg total RNA (AM1931, Thermo Fisher Scientific, Netherlands), followed by treatment with DNAse I and silica-membrane purification (74,134, Qiagen, Germany). RNA was quantified using the Qubit RNA HS Assay Kit (Q32852, Thermo Fisher Scientific) and diluted to a concentration of 100 ng/μl. Confirm the quality of total RNA using the Fragment Analyzer (Advanced Analytical Technologies, Germany). For samples with an RNA Quality Number (RQN) greater than or equal to 6, RNA library preparation will be conducted using protocols certified under ISO/IEC 17025 (TruSeq RNA Library Preparation Kit v2, Illumina, United States). After cDNA synthesis of enriched oligo (dT) beads and fragmented mRNA, adapter ligation and PCR amplification are performed. Sequencing with Illumina HiSeq 2500v4 sequencer using a 126 bp paired-end sequencing strategy. According to the manufacturer’s protocol, each sample should be sequenced to a minimum depth of 12.5 Gbp, resulting in approximately 50 million read pairs. Perform image analysis, base calling, and quality control using Illumina data analysis pipeline RTA v1.18.64 and Bcl2fastq v1.8.4. RNAseq reads are provided in compressed Sanger FASTQ format.

Each input sample file is analyzed by FastQC (version 0.10.1). Known overexpressed sequences detected by FastQC were subjected to adapter trimming using cutadapt (version 1.2), with a minimum accepted read length set at 20 bp. Next, use a sickle (version 1.33) to trim low-quality bases. Each read is synchronized through a custom Python script to maintain correspondence and merged into a single file. Use the GSNAP alignment tool (version 2014–12–23) to align reads against the human reference genome GRCh38, with the options set for novel splicing discovery (−-novel splicing 1) and unique alignment (−-paths 1 and --quiet-if-excessive). The output SAM file is compressed, position sorted, and indexed using the Picard suite (version 1.141). Differentially expressed genes between the control group, model group, and classical ketogenic diet group samples were filtered using “Limma” in Python script (with thresholds of |logFC| > 1 and *p*-value <0.05). Use Python script to draw the heatmap of differentially expressed genes, and also draw the volcano plot of differentially expressed genes and perform GO and KEGG enrichment analysis (Saeidian et al., 2020; Arindrarto et al., 2021; Wang et al., 2021).

### 2.9 RT-qPCR

Extract hippocampal tissues from the control, Model, and classic ketogenic diet group mice. Extract total RNA from hippocampal tissue using TRIzol reagent (15,596,026, ThermoFisher, United States). Reverse transcribe 1 μg of RNA into cDNA using oligo (dT) primer. The primer sequence can be found in Supplementary Table S1, and Shanghai Biochemical Co., Ltd. synthesized it Synthesize cDNA using Maxima Reverse Transcriptase (EP0751, Thermo Scientific). Using SYBR Green (K0222, Thermo Scientific) for RT-qPCR detection, three replicates were set for each sample. GAPDH serves as an internal reference. Calculate relative expression using the 2^−ΔΔCt^ method (Liang et al., 2017; Mazdeh et al., 2018; Wang et al., 2021).

### 2.10 Western blot

The hippocampus tissue was digested in RIPA buffer (Thermo Scientific, United States). Protein concentration was determined using the BCA (Bicinchoninic Acid) method, and quantification was performed with the BCA1 assay kit (Sigma-Aldrich, United States). The samples are separated on SDS/PAGE and transferred to a PVDF membrane. Incubate the membrane in TBS buffer containing Tween-20 and 5% skim milk for 1 h. Incubation with the following antibodies was performed overnight at 4°C: MA5-32245 (VAV2, dilution 1:1000), PA5-35382 (ADCY3, dilution 1:2000), MA3-920 (CACNA1S, dilution 1:500), PA5-104261 (CALLM3, dilution 1:1000), MA1-157 (PRKACA, dilution 1:1000), PA5-104261 (GRIN2B, dilution 1:1000), MA1-083 (CREB), and BFNE84953 (pCREB). Incubate the membrane after washing, and then incubate with the HRP-conjugated secondary antibody (ab6721, Abcam, United Kingdom) in the same TBS buffer for 1 h. Wash the membrane with TBST for 5 min; repeat 3 times. For development, protein quantification analysis was performed using the Genesys imaging system (Gene Company Limited, UK) and ImageJ 1.80u software. Protein quantification analysis is performed by calculating the ratio of each protein’s grayscale value to the internal reference’s grayscale value, GAPDH (Zou et al., 2016; Wang et al., 2021). Repeat the experiment three times each time.

### 2.11 ELISA

Homogenized samples of mouse hippocampal tissue were collected, and the expression levels of cAMP (ab290713, from the United Kingdom) and PKA (JK-E3114, from Crysto Biotech in China) were detected using an enzyme-linked immunosorbent assay (ELISA) kit following the manufacturer’s instructions (Ding et al., 2014; Ma et al., 2014).

### 2.12 Electrophysiological analysis

Mice were anesthetized with 0.5% pentobarbital sodium for brain extraction. The brain was quickly removed and placed in artificial cerebrospinal fluid (ACSF) for cooling. The ACSF composition consisted of: choline chloride 110 mM, NaHCO_3_ 26 mM, d-glucose 10 mM, Na-ascorbate 11.6 mM, MgCl_2_ 7 mM, Na-pyruvate 3.1 mM, KCl 2.5 mM, NaH_2_PO_4_ 1.25 mM, and CaCl_2_ 0.5 mM (Wickham et al., 2020). The VT-1000S vibrating microtome (Leica, Germany) cut longitudinal hippocampal slices (300 μm) in ACSF and transferred them to a regular ACSF storage chamber. The slices were maintained at a constant temperature of 32°C for 30 min before recording, followed by an additional hour at room temperature. All solutions are saturated with 95% O_2_/5% CO_2_ (vol/vol). Place the brain slices in the recording chamber and perfuse with ACSF at a 2 mL/min flow rate. Perform whole-cell patch clamp recordings on CA1 pyramidal neurons using an upright microscope with an infrared-sensitive CCD camera (DAGE-MTI, IR-1000E). The resistance value of the pipette is 3–5 MΩ, and the micropipette puller (P-97, Sutter instrument) is used for pulling. The recording was performed using the MultiClamp 700B amplifier and the 1440A digitizer (Molecular Devices). Recorded spontaneous inhibitory postsynaptic currents (sIPSCs) were maintained at −70 mV in the presence of 20 μM CNQX and 100 μM AP-5, and the pipette solution contained (in millimolar): 140 mM CsCl, 10 mM Hepes, 0.2 mM EGTA, 1 mM MgCl2, 4 mM Mg-ATP, 0.3 mM Na-GTP, 10 mM phosphocreatine, and 5 mM QX314 (pH 7.40, 285 mm). Measurements of evoked inhibitory postsynaptic currents (PSCs) were conducted with the stimulating electrode placed on the Schaffer Collaterals (SC)-CA1 pathway, approximately 100 micrometers away from the recording pipette (Wang et al., 2021).

### 2.13 Calcium imaging technology

Separate and culture neurons from the mouse brain in suspension in a culture medium. Then, add 1 microliter of Fluo4-AM (Beyotime.S1060) to 1 milliliter of DMEM solution and incubate the neurons for 30 min. Remove the Fluo4-AM solution, wash three times with HBSS solution (14170112), excite with fluorescence dye at 480 ± 15 nanometers, and measure fluorescence emission at 535 ± 25 nanometers. Capture images every 2 s using a digital camera (Nikon Corporation, Japan) and analyze them using ImageJ software (Ait Ouares et al., 2020; Yang et al., 2020). The fluorescence intensity of Fluo-4 was quantified in a minimum of 5 fields, each containing at least 100 cells. The average fluorescence intensity was computed for each field. Following this, the average fluorescence intensity of the control group (oe-NC or KD group) in every experiment was normalized to 100%. Subsequently, the relative fluorescence intensity of calcium ions in each experimental group was determined by calculating the ratio of its average fluorescence intensity to that of its respective control group (Wang et al., 2013).

### 2.14 Blood glucose and blood ketone monitoring

Measure the concentrations of blood ketones (β-hydroxybutyrate) and glucose using Abbott’s blood glucose and ketone monitoring system (FreeStyle Optium Neo124434). According to the manufacturer’s instructions, disinfect the mouse tail with 70% ethanol, then use clean scissors to cut off the tip and collect a drop of blood. Use Abbott test strips to measure levels of β-hydroxybutyrate or glucose (Wang et al., 2021).

### 2.15 Recruitment and other processing of clinical samples

This study involved a 6-month clinical observation of 60 epilepsy patients admitted to our hospital between January and June 2023. This study has been approved by the Clinical Ethics Committee of our hospital, and written informed consent has been obtained from the patients and their families, strictly adhering to the Helsinki Declaration. Inclusion criteria include: (1) Meeting the diagnostic criteria for epilepsy, including primary epilepsy, secondary epilepsy, focal epilepsy, and generalized epilepsy; (2) No improvement after treatment with two or more anticonvulsants drugs; (3) Age older than 3 years; (4) The study has obtained approval from the medical ethics committee. Exclusion criteria include: (1) individuals with disorders of lipid metabolism; (2) individuals with severe cardiovascular, pulmonary, renal, and hematologic diseases; (3) individuals with severe hepatic and renal impairment; (4) individuals with mental disorders, behavioral disorders, and language disorders; (5) pregnant women; (6) individuals who have used a ketogenic diet or had changes in anticonvulsants treatment in the past 12 months; (7) individuals who have experienced status epilepticus in the previous 6 months or have undergone surgical resection or vagus nerve stimulator implantation in the previous 12 months; (8) individuals with other conditions prohibiting the use of a ketogenic diet.

Eligible patients were randomly assigned into two groups using a computer-generated random number list: the Conventional Diet Group (CAU) and the Classic Ketogenic Diet Group (KD), each consisting of 30 individuals. The KD group comprised 12 males and 18 females with a mean age of 14.53 years, while the CAU group included 10 males and 20 females with a mean age of 13.73 years (Neal et al., 2009; Sondhi et al., 2020).

Both groups underwent a one-week baseline observation period, during which each patient recorded detailed medical history and underwent examinations, including measurement of β-hydroxybutyrate, cholesterol, triglycerides, high-density lipoprotein, and low-density lipoprotein levels. The type, frequency, age of onset, family history, developmental status, and treatment history of epilepsy were also recorded. During hospitalization, participants in the diet group received dietary guidance, and family members collected data after patients were discharged. The CAU group received conventional anticonvulsants treatment and standard diet, while the KD group received adjunctive ketogenic diet treatment based on the CAU group’s treatment. During the ketogenic diet therapy, daily meals should be prepared according to the principles of the KD diet. The recommended ratio of fats to proteins and carbohydrates is 4:1, with protein intake meeting the minimum requirement suggested by the World Health Organization (WHO). Adequate supplementation of sugar, calcium, and vitamins is also essential. For patients during the ketogenic diet therapy, assess the contraindications and adverse reactions of the ketogenic diet therapy, and evaluate the effectiveness of epilepsy treatment based on the grading criteria of the International League Against Epilepsy (ILAE). The standard is divided into the following four levels: (1) Complete epilepsy control: patients have not experienced any epilepsy for two consecutive years and are not using any anticonvulsants drugs; (2) improvement: patients have a reduction in epilepsy frequency of 75% or more; (3) Some improvement: patients have a reduction in epilepsy frequency of 50 to 74%; (4) Ineffective: patients have a reduction in epilepsy frequency of less than 50%, or there is no improvement in symptoms (Neal et al., 2009; Lambrechts et al., 2017; Kverneland et al., 2018).

During the 6 months of the study, the epilepsy frequency and duration of epileptic patients in the CAU and KD groups were assessed through observation and recording by family members or caregivers. Additionally, regular examinations, such as electroencephalograms, were conducted.

Before and after treatment, we collected peripheral venous blood samples (3 mL) from the patients and measured the levels of β-hydroxybutyrate, triglycerides (TG), cholesterol (HyperChol), high-density lipoprotein (HDL), and low-density lipoprotein (LDL).

Obtain 3 mL of a peripheral venous blood sample using venipuncture into an EDTA preparation tube, and detect the expression levels of ADCY3 (ADCY3, RENJIE), cAMP (ab290713, United Kingdom), and PKA (CB10491-Hu, SCI-BIO) in the sample using enzyme-linked immunosorbent assay (ELISA) kits.

### 2.16 Statistical analysis

Using the fixed effects model, a Meta-analysis was conducted on direct evidence using R 4.3.0 software and the Meta package. The treatment efficacy of the ketogenic diet in patients with epilepsy was directly compared, and the respective odds ratios (OR) and 95% confidence intervals (CI) for each study were combined to obtain the overall effect size. In addition, Bayesian statistical methods are used for network meta-analysis, comparing the treatment effects through indirect evidence with corresponding risk ratios (RR) and 95% confidence intervals. Create a probability graph of the levels to rank these intervention measures and compare them to choose the best therapeutic effect. The I2 test is used to analyze heterogeneity among different studies. A fixed-effect model is adopted when *p* ≥ 0.05 and I2 < 50%. A random-effect model is adopted when *p* < 0.05 and I2 > 50%.

The data was statistically processed using SPSS 26.0 software. The continuous variable is expressed as χ ± s. The comparison between two groups was conducted using a t-test, while the comparison among multiple groups was performed using a one-way analysis of variance. The categorical variable was analyzed using the chi-square test. Count data is presented as rates or percentages. A significance level of *p* < 0.05 indicates a statistical difference.

## 3 Results

### 3.1 The classical ketogenic diet has therapeutic effects in treating epilepsy

The ketogenic diet is a high-fat, low-carbohydrate diet widely used in treating patients with epilepsy. There are several types of ketogenic diets, including the classic ketogenic diet (KD), medium-chain triglyceride ketogenic diet (MCT), modified ketogenic diet, and a low-calorie ketogenic diet (MAD) (Sampaio, 2016; Martin-McGill et al., 2020; Sondhi et al., 2020). The individual variances in patients with epilepsy suggest that their responses to different treatments may vary. A detailed comparison of various ketogenic diets can offer clinicians more specific guidance to assist them in selecting the most suitable dietary treatment plan for each patient based on their specific circumstances (van der Louw et al., 2016).

First, we searched English databases such as PubMed, Cochrane Library, Embase, and Web of Science (Sourbron et al., 2020; Yang et al., 2022). We obtained a total of 1,211 articles, excluding 251 duplicated articles. We then screened out 411 articles, including animal experiment articles, review articles, cohort study articles, and irrelevant articles, by reading the titles and abstracts. Next, we read the full texts of the remaining 122 articles and eventually included eight randomized controlled trial articles (Supplementary Figure S1), totaling 692 patients. Among them, 4 studies compared the ketogenic diet with the conventional diet (CAU), with 111 patients in the ketogenic diet group and 114 patients in the conventional diet group. The detailed characteristics are shown in Supplementary Table S2. Additionally, four studies compared different types of ketogenic diets, including 467 patients. Detailed characteristics are also shown in Supplementary Table S2. Quality assessment for the included 8 literature was conducted (risk bias), and the Jadad score ranged from 4 to 7, indicating all studies were of high quality (Supplementary Table S3). The Cochrane risk of bias assessment results are shown in Supplementary Figure S2.

**Figure 1:**
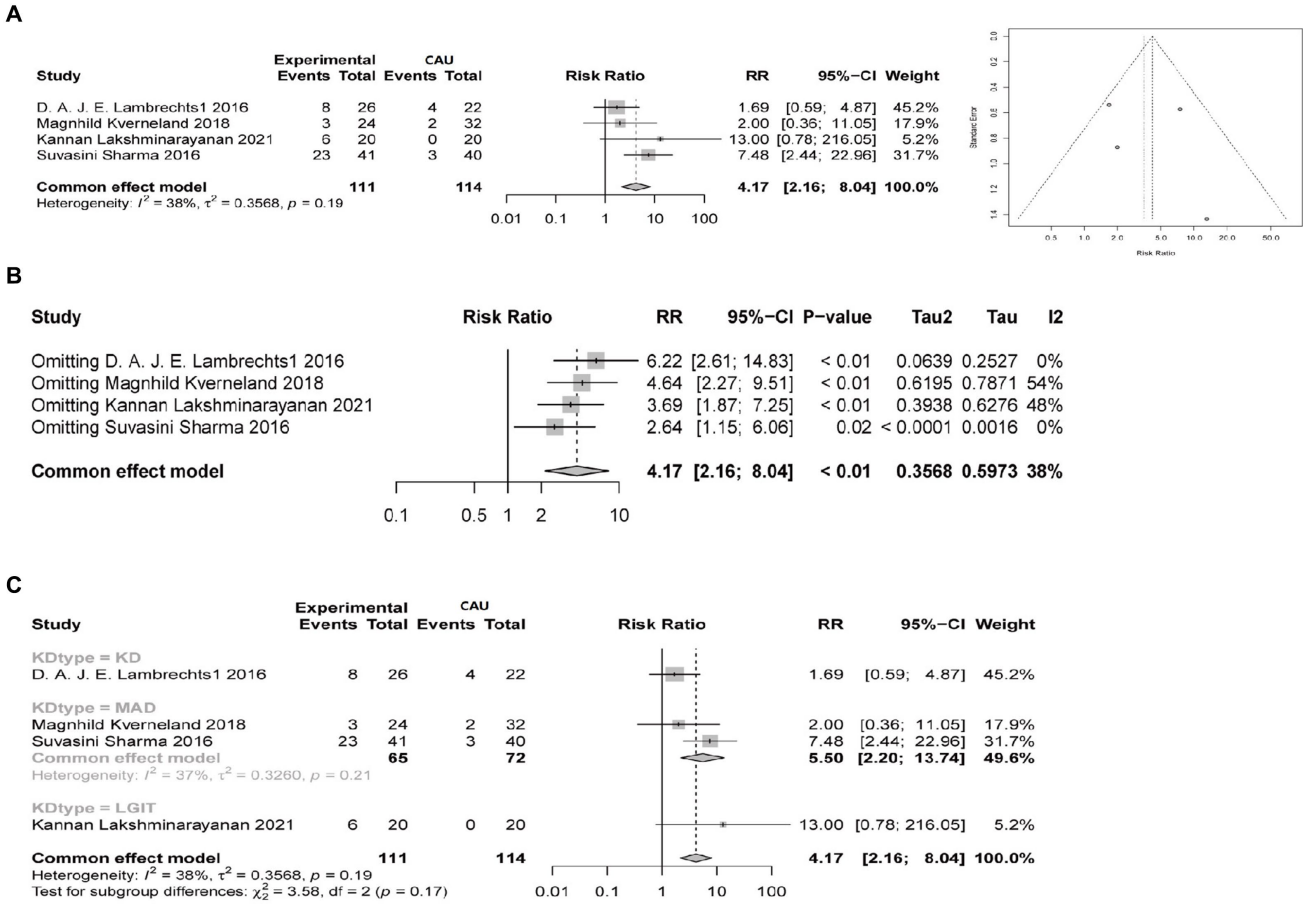
Comparison of the therapeutic efficacy between the ketogenic diet and conventional diet control in reducing epilepsy (≥50% reduction in epilepsy). Experimental group refers to the group on a ketogenic diet, with a total sample size of 111 cases, while CAU represents the control group on a conventional diet, with a total sample size of 114 cases. Events indicate the number of cases with a ≥ 50% reduction in epilepsy. RR indicates the relative risk ratio, where RR > 1 indicates that the number of cases with a ≥ 50% reduction in epilepsy in the ketogenic diet group is multiplied by the ratio compared to the control group on a conventional diet. The 95% confidence interval (95%-CI) reflects the possible range of effect sizes to support the results. I2 is used to evaluate the magnitude of heterogeneity between studies, with I2 < 50% indicating an acceptable level of heterogeneity. **(A)** Comparison of the therapeutic efficacy of ketogenic and conventional diets in reducing epilepsy by at least 50% at 3 months, with a funnel plot to assess publication bias. **(B)** Sensitivity analysis of the therapeutic efficacy of ketogenic and conventional diets in controlling epilepsy at 3 months, evaluating the source of heterogeneity. **(C)** Subgroup analysis of different types of ketogenic diets to evaluate the heterogeneity of the studies.

To verify the efficacy of the ketogenic diet in treating epileptic epilepsy, we analyzed four clinical studies comparing the conventional diet (CAU) with different types of ketogenic diets (KD, MCT, MAD, LGIT), evaluating a total of 225 patients. In these four studies, the ketogenic diet was used as an adjunctive therapy for epileptic epilepsy. In these four studies, the efficacy rate of a 3-month ketogenic diet treatment was 36.0% (40/111). The I2 test showed no heterogeneity between the groups (I2 = 38%, *p* = 0.19), and the funnel plot showed no obvious publication bias (Figure 1A). When conducting sensitivity analysis, a fixed effects model was used. The results showed that Magnhild Kverneland had an I2 of 54%, indicating slight heterogeneity. The overall pooled risk ratio (RR) was 4.17, with a 95% confidence interval (CI) of [2.16, 8.04]. The *p*-value was less than 0.05, indicating a statistical difference (Figure 1B). It suggests that the ketogenic diet is superior to the conventional diet in treating epilepsy. Because the ketogenic diet has 4 different types, we conducted subgroup analysis on different types of ketogenic diet subgroups to assess the effectiveness of the ketogenic diet and the heterogeneity of the studies. The results show that when comparing different ketogenic diets with a conventional diet group, the I2 value is less than 50%, indicating acceptable heterogeneity. The relative risk ratio between the KD and CAU groups was 1.69, 95% CI = 1.69[0.59, 4.87], with no statistical difference. The merged relative risk ratio between the MAD group and the CAU group was 5.50, 95% CI = [2.20, 13.74], I2 = 37%, *p* = 0.21, indicating an improvement in treatment effect in the MAD group compared to CAU, but with no statistical difference. The relative risk ratio between the LGIT and CAU groups was 13, 95% CI = [0.78, 216.05], with no statistical difference (Figure 1C).

**Figure 2:**
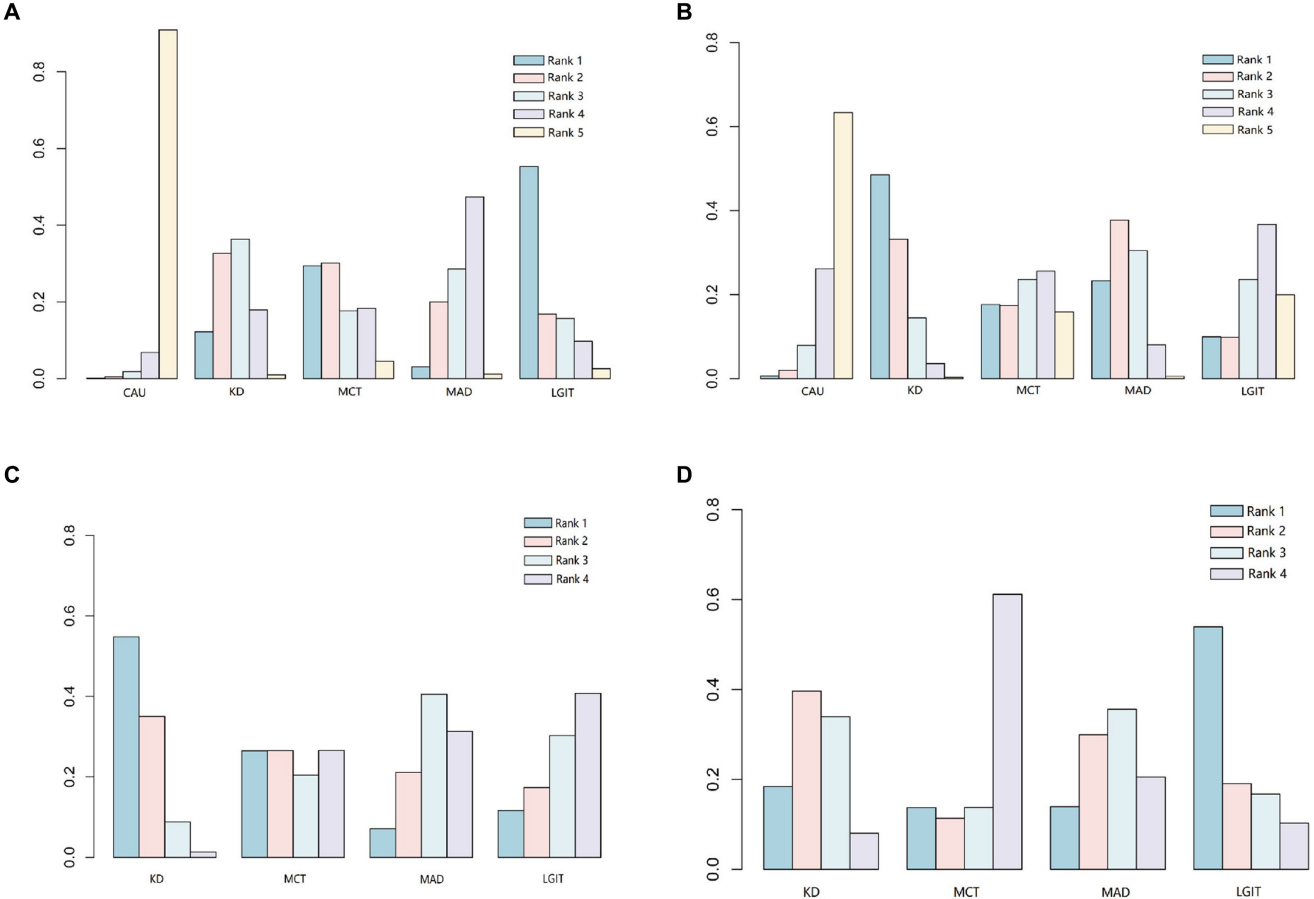
Comparison of the probabilities of different types of ketogenic diets at various levels. CAU refers to the control group on a conventional diet, KD represents the group on a classic ketogenic diet, MCT refers to the group on a medium-chain triglyceride ketogenic diet, MAD stands for the group on a modified Atkins diet, and LGIT describes the group on a low glycemic index ketogenic diet. Rank indicates the ranking probability of different types of ketogenic diets at specific levels (1–5). The levels indicate the likelihood of being the best treatment, second-best treatment, and so on, with Level 1 being the best and Level 5 being the worst. **(A)** Comparison of the therapeutic efficacy of different ketogenic diets in reducing epilepsy by at least 50% at 3 months. **(B)** Comparison of the therapeutic efficacy of different ketogenic diets in reducing epilepsy by at least 90% at 3 months. **(C)** Comparison of the therapeutic efficacy of different ketogenic diets in reducing epilepsy by at least 50% at 6 months. **(D)** Comparison of the therapeutic efficacy of different ketogenic diets in reducing epilepsy by at least 90% at 6 months.

To compare the differences in the therapeutic effects of different types of ketogenic diets on epilepsy, a network meta-analysis was conducted on the included 8 studies. The therapeutic effects of different ketogenic diets are represented as relationships between nodes in the network graph (Supplementary Figure S3). The greater the number of nodes, the higher the degree of association. The study, analyzing the reduction of epilepsy by 50% in 3 months (including ①②④⑤⑦⑧), found that the ketogenic diet (KD) and modified Atkins diet (MAD) were superior to conventional anticonvulsants drugs (CAU) (Supplementary Table S4; Supplementary Figure S4A). The probability graph suggests that LGIT is better than KD, MCT, and MAD, and KD is better than MAD (Figure 2A). The study analyzing three months of reduced epilepsy by 90% (including ①④⑤⑦⑧) found that KD was superior to CAU (Supplementary Table S5; Supplementary Figure S4B). The level probability graph indicates that KD is superior to CAU (Figure 2B). The study analyzing a 50% decrease in epilepsy over 6 months (including ⑤⑥⑦) found that the KD was superior to CAU (Supplementary Table S4; Supplementary Figure S4C). MCT, MAD, and LGIT were superior to the CAU group, but the differences were insignificant. The probability graph indicates that the KD group has the best effect (Figure 2C; Supplementary Figure S4B). The study, which analyzed a 90% reduction in epilepsy over 6 months (including ⑤⑥⑦), found that KD, MAD, and LGIT were superior to MCT, while KD and LGIT were superior to MAD (Supplementary Table S5; Supplementary Figure S4D). The difference is not statistically significant, and the rank probability plot indicates that the KD group is superior to the MCT, MAD, and LGIT groups (Figure 2D). The meta-analysis results indicate more robust support for using the conventional ketogenic diet in treating epileptic epilepsy.

### 3.2 Transcriptome sequencing indicates that the ketogenic diet exerts its therapeutic effect on epilepsy by activating the cAMP signaling pathway

**Figure 3:**
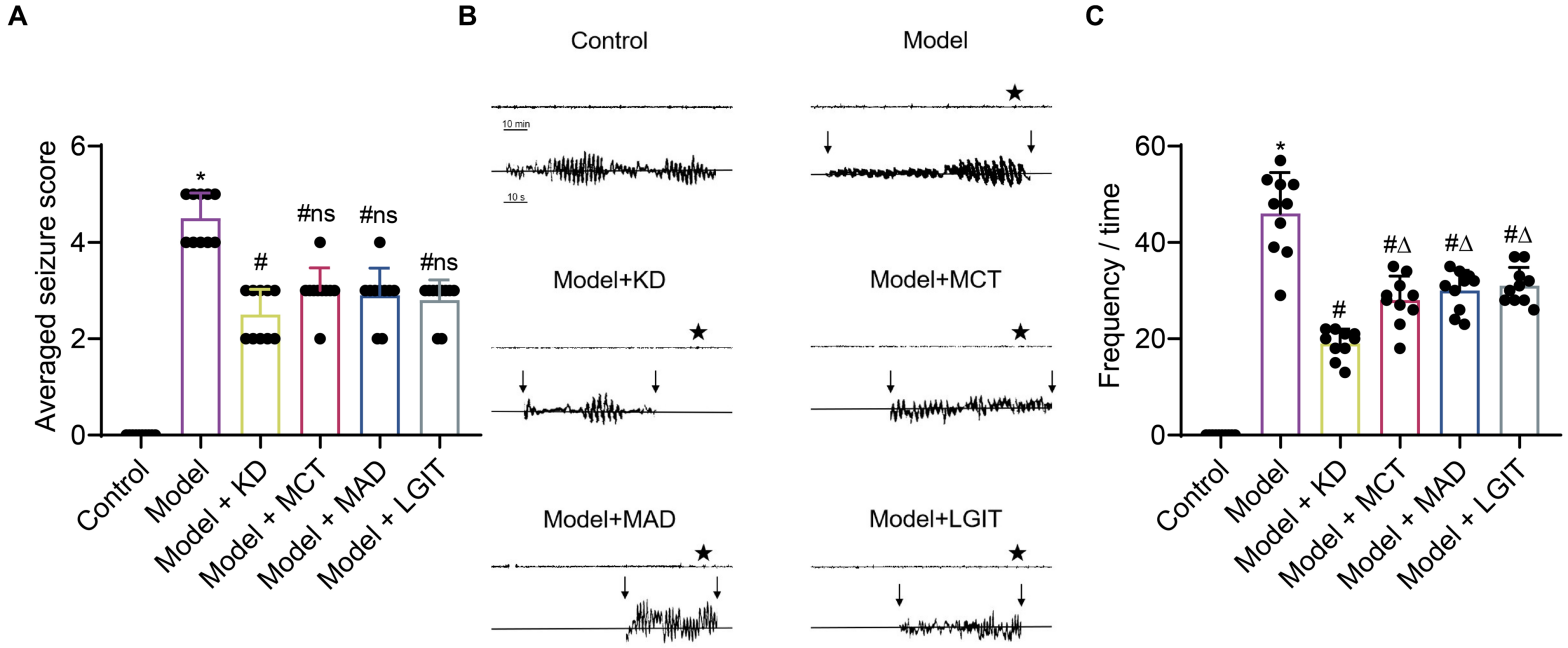
Analysis of the therapeutic efficacy of a classic ketogenic diet in controlling epilepsy. Control refers to the standard group, Model represents the epilepsy model group, KD stands for the group on a classic ketogenic diet, MCT refers to the group on a medium-chain triglyceride ketogenic diet, MAD stands for the group on a modified Atkins diet, and LGIT describes the group on a low glycemic index ketogenic diet. Each group had a sample size of n = 10. **(A)** Racine scoring, *denotes a difference compared to the Control group, # denotes a difference compared to the Model group, *p* < 0.05, ns denotes no difference compared to the KD group, *p* > 0.05. **(B)** In EEG analysis of epilepsy controlled by the ketogenic diet, vertical arrows indicate the start and end of epilepsy in mice, the Control group had no epilepsy activity, the Model group represents the baseline epilepsy activity in mice, KD, MCT, MAD, and LGIT groups showed a decrease in epilepsy activity wave amplitude compared to the Model group. **(C)** Comparison of epilepsy frequency in mice with epilepsy, *denotes a difference compared to the Control group, # denotes a difference compared to the Model group, *p* < 0.05, ∆ denotes a difference compared to the KD group, *p* < 0.05.

To further validate the results of the aforementioned meta-analysis, this study constructed a chronic epilepsy mouse model and utilized video monitoring to observe spontaneous recurrent seizures in the mice. The results showed that the Racine scoring system was used to assess motor epilepsy activity in epileptic patients. Compared with the Control group, the Model group showed increased epilepsy scores. Compared with the Model group, the group using KD, MCT, MAD, and LGIT, four types of ketogenic diets, showed reduced epilepsy scores (Figure 3A). The EEG results indicated that the amplitude of epilepsy activity in the Model group was increased compared to the Control group. Compared with the Model group, mice using KD, MCT, MAD, and LGIT ketogenic diets reduced the amplitude of epileptic epilepsy activity (Figure 3B). Compared to the Control group, the epilepsy frequency in the Model group increased. Compared with the Model group, the groups using KD, MCT, MAD, and LGIT ketogenic diets demonstrated a decrease in the frequency of epileptic epilepsy. Comparing the frequency of epileptic epilepsy among different types of ketogenic diets, it can be observed that the ketogenic diet (KD) is superior to the medium-chain triglyceride diet (MCT), the modified Atkins diet (MAD), and the low glycemic index treatment (LGIT) (Figure 3C), which is consistent with the results of the meta-analysis.

**Figure 4:**
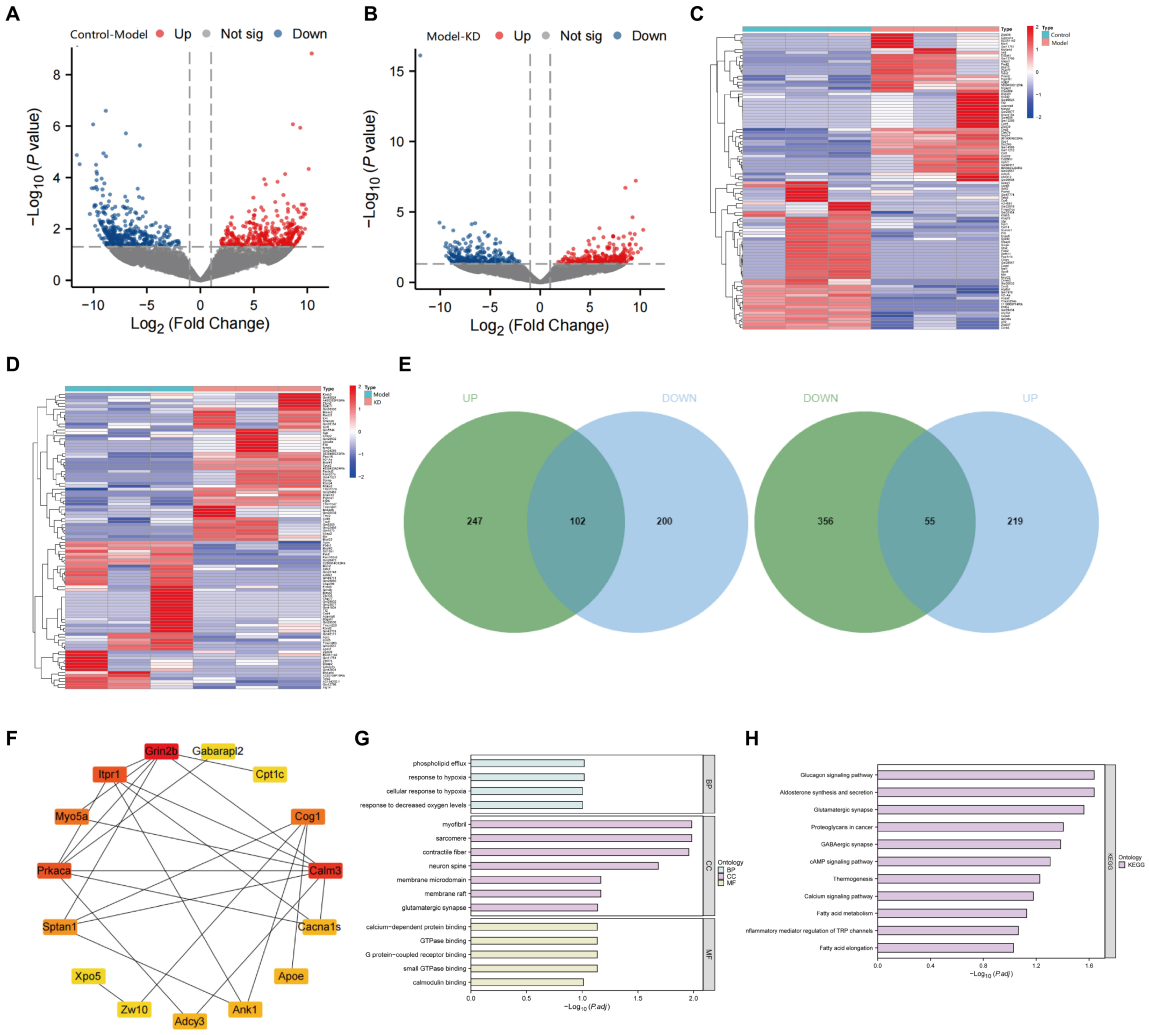
Transcriptome sequencing analysis results. Control refers to the standard group, the Model represents the epilepsy model group, and KD stands for the group on a classic ketogenic diet. Each group had a sample size of *n* = 3. **(A)** Comparison of the Model group with the Control group, red dots indicate upregulated gene expression, green dots indicate downregulated gene expression, the *p*-value represents the significance of differential gene expression, and a higher Log2FC (log2 fold change) indicates a more difference in expression. **(B)** Comparison of the epilepsy model group with the classic KD group, *p* < 0.05, indicating a difference. Red dots indicate upregulated gene expression, green dots indicate downregulated gene expression, the p-value represents the significance of differential gene expression, and a higher Log2FC (log2 fold change) indicates more expression differences. **(C)** Hierarchical clustering analysis of differentially expressed genes between the Control and Model groups. **(D)** Hierarchical clustering analysis of differentially expressed genes between the Model and KD groups. **(E)** Venn diagrams depicting the intersection of upregulated genes in the Model group (compared to the Control group) with downregulated genes in the KD group (upper diagram), and the intersection of downregulated genes in the Model group (compared to the Control group) with upregulated genes in the KD group (lower diagram). Green represents genes differentially expressed between the Control and Model groups, blue represents genes differentially expressed between the Model and KD groups, and the overlapping area indicates intersecting genes. **(F)** Protein interaction analysis among the Control, Model, and KD groups. **(G)** GO enrichment analysis. **(H)** KEGG pathway enrichment analysis.

To investigate the potential mechanisms of ketogenic diet therapy in treating epileptic epilepsy, we performed transcriptome sequencing on the Control group, Model group, and KD group. The results indicate that volcano plots can provide an overall view of the distribution of differential genes (Figures 4A,B). The Model group obtained 5,054 upregulated differentially expressed genes and 5,497 down-regulated differentially expressed genes compared with the Control group (Figure 4A). Compared to the Model group, the KD group had 4,693 upregulated differentially expressed genes and 5,038 downregulated differentially expressed genes (Figure 4B). Differential gene expression analysis was conducted using the criteria |LogFC| > 1 and *p* < 0.05 as selection standards (Figures 4C,D). Genes with similar or identical expression patterns were grouped for analysis, where high gene expression levels are represented in red and low levels in blue. By intersecting the differentially expressed genes between the Control and Model groups with those between the Model and KD groups but in opposite trends, a total of 157 intersecting genes were identified (Figure 4E). Protein–protein interaction analysis was conducted on the 157 intersecting genes encoded by these proteins. The top 15 genes, sorted by degree value, are GRIN2B, CALM3, PRKACA, ITPR1, COG1, MYO5A, SPTAN1, ADCY3, ANK1, APOE, CACNA1S, ZW10, CPT1c, GABARAP12, XPO5 (Figure 4F).

The results of GO and KEGG enrichment analysis showed that there were enrichments in biological processes such as response to hypoxia, response to decreased oxygen levels, cellular response to hypoxia, and phospholipid efflux (Figure 4G), as well as in signaling pathways, including Proteoglycans in cancer, cAMP signaling pathway, Calcium signaling pathway, Glucagon signaling pathway, Glutamatergic synapse, GABAergic synapse, and Fatty acid elongation (Figure 4H). After consulting, it was found that the genes enriched in the cAMP signaling pathway were mainly VAV2, CALM3, ADCY3, CACNA1S, PRKACA, and GRIN2B. Among them, 5 genes overlapped with the sorted degree values, suggesting that KD may regulate the cAMP signaling pathway in treating epilepsy by modulating the above genes.

**Figure 5:**
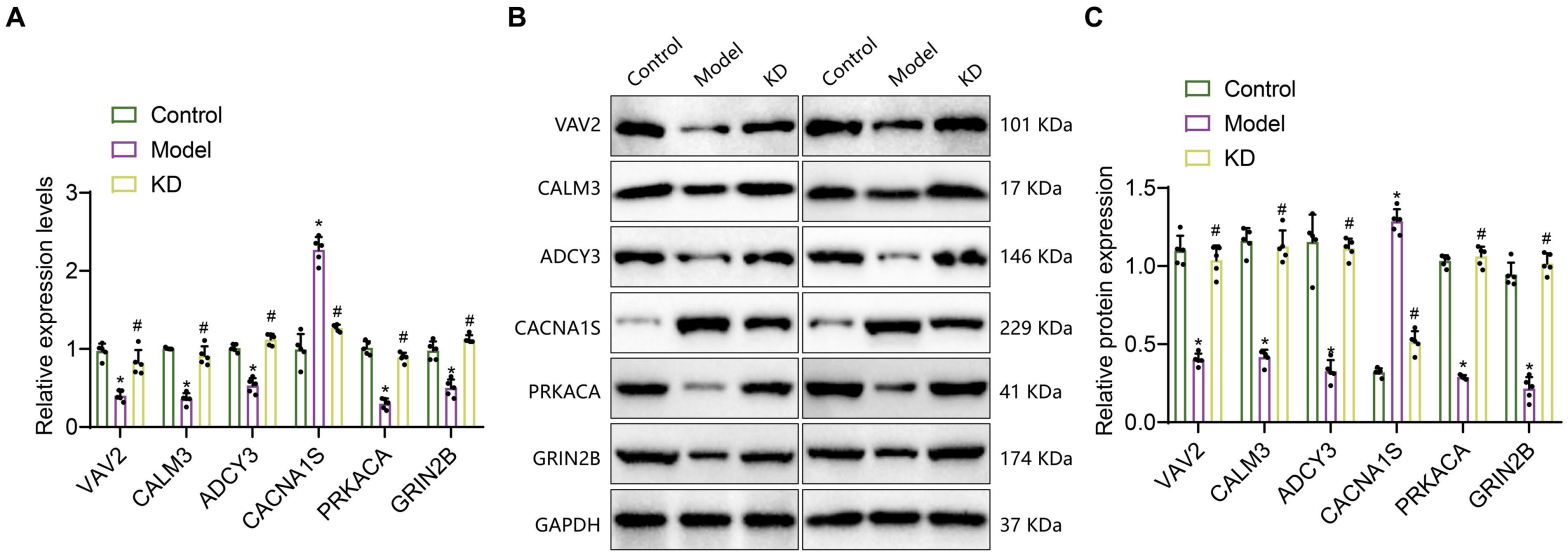
The expression of 6 critical genes in the hippocampal tissue of epileptic mice. Control refers to the standard group; Model refers to the epileptic model group; KD refers to the classic ketogenic diet group. The sample size in each group was *n* = 5. **(A)** RT-qPCR analysis, * indicates the difference between the Model group and the Control group (*p* < 0.05); # indicates the difference between the KD group and the Model group (*p* < 0.05); **(B,C)** Western Blot analysis, * indicates the difference between the Model group and the Control group (*p* < 0.05); # indicates the difference between the KD group and the Model group (*p* < 0.05).

Transcriptome sequencing results indicate that, compared with the Control group, the expression levels of VAV2, CALM3, ADCY3, PRKACA, and GRIN2B are down-regulated, while the expression level of CACNA1S is upregulated. Compared with the Model group, the expression levels of VAV2, CALM3, ADCY3, PRKACA, and GRIN2B were upregulated, while the expression level of CACNA1S was downregulated. To further validate the transcriptome data, we performed RT-qPCR and Western blot experiments to detect the mRNA and protein expression levels of VAV2, CALM3, ADCY3, CACNA1S, PRKACA, and GRIN2B. As shown in Figures 5A–C, the results showed that compared to the Control group, the mRNA and protein levels of VAV2, CALM3, ADCY3, PRKACA, and GRIN2B were downregulated, while the mRNA and protein levels of CACNA1S were upregulated. Compared with the Model group, the mRNA and protein levels of VAV2, CALM3, ADCY3, CACNA1S, PRKACA, and GRIN2B were upregulated, while the mRNA and protein levels of CACNA1S were downregulated, which is consistent with the expression trend observed in the transcriptome sequencing results.

The above results suggest that these six genes, VAV2, CALM3, ADCY3, CACNA1S, PRKACA, and GRIN2B, may be critical genes influencing epileptic epilepsy. KD may improve epileptic epilepsy by regulating the expression of VAV2, CALM3, ADCY3, CACNA1S, PRKACA, and GRIN2B, thereby modulating the cAMP signaling pathway and inhibiting neuronal activity.

### 3.3 ADCY3 activation improves epilepsy onset in epilepsy through the cAMP signaling pathway

To explore the possible mechanisms of the involvement of critical genes such as VAV2, CALM3, ADCY3, and CACNA1S in epileptic epilepsy, we have consulted relevant literature. We found that ADCY3 (adenylate cyclase 3) is an activator of cAMP. Increased expression of ADCY3 can catalyze ATP to generate cAMP, which then binds to the regulatory subunits of PKA, activating the catalytic subunits of PKA and subsequently activating PKA (Hong et al., 2013; Zheng et al., 2023). Therefore, regulating the activity of cAMP plays a crucial role in controlling the excitability of neurons and networks (Zhang et al., 2021b). Based on this, we found that the remaining four genes are downstream in the cAMP signaling pathway, and their expression may be closely associated with cAMP activation.

Research has found that activation of the cAMP signaling pathway in the hippocampal neurons of brain tissue has a neuroprotective effect (Chen et al., 2021; Essam and Kandil, 2023). The expression levels of the ADCY3 gene increase during dietary restriction and ketone body intake (Zhang et al., 2017; Goni et al., 2018). Therefore, ADCY3 may be a potential target for the treatment of epilepsy.

Several studies have indicated that cAMP exhibits dual regulatory effects. It enhances the strength of excitatory neural circuits and, simultaneously, acts locally on postsynaptic GABA receptors to reduce inhibitory synaptic plasticity (Lee, 2015). In the context of epilepsy research, activation of the cAMP-PKA pathway has been shown to have inhibitory effects during seizures (Qi et al., 2019). Furthermore, Forskolin, a adenylyl cyclase activator, has been demonstrated to increase cAMP levels when administered subcutaneously in mice, thereby preventing epileptic seizures (Sano et al., 1984). The findings of these studies align with those of the present study, suggesting that increasing cAMP has antiepileptic effects in the regulation of epilepsy.

To investigate the activation of the ADCY3 in the cAMP signaling pathway and its impact on epileptic seizures (Hosseini-Zare et al., 2011), we established an epileptic mouse model and conducted experimental validation. The experiments were divided into four groups: oe-NC (overexpression lentivirus control group), oe-ADCY3 (overexpression lentivirus ADCY3 group), oe-ADCY3 + DMSO (overexpression lentivirus ADCY3 group with intraperitoneal injection of varying doses of DMSO), and oe-ADCY3 + RMI 12330A (overexpression lentivirus ADCY3 group with intraperitoneal injection of varying doses of cAMP inhibitor, RMI 12330A).

**Figure 6:**
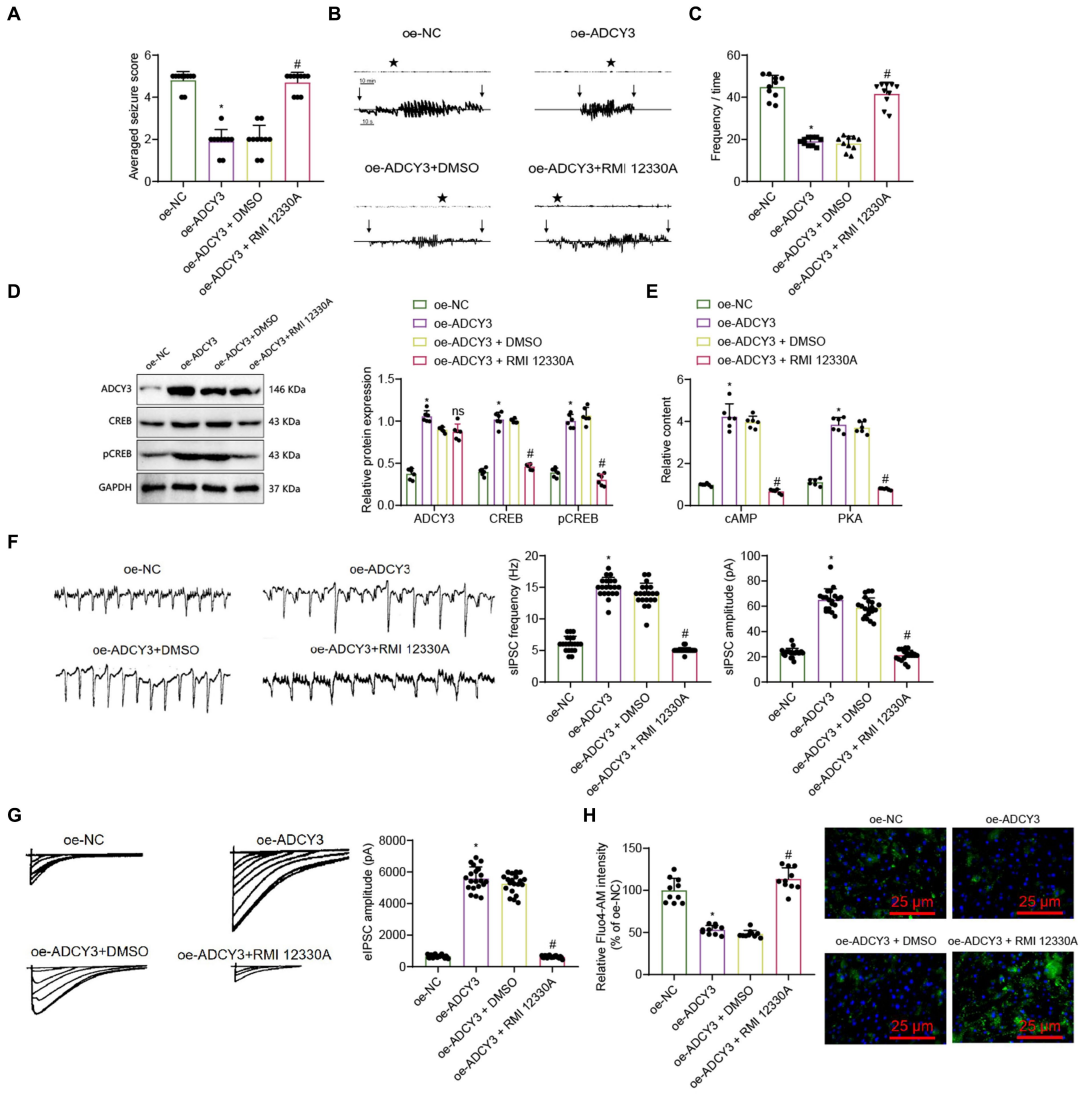
Activation of the cAMP signaling pathway by ADCY3 improves epileptic epilepsy. **(A)** Behavioral observation in mice (Racine score), *n* = 10, *indicates difference between the oe-ADCY3 group and the oe-NC group (*p* < 0.05), # indicates difference between the oe-ADCY3 + RMI 12330A group and the oe-ADCY3 + DMSO group (*p* < 0.05). **(B)** Analysis of mouse EEG, Vertical arrows indicate the beginning and end of seizures in mice. **(C)** Frequency of epileptic epilepsy, *n* = 10, *indicates difference between the oe-ADCY3 group and the oe-NC group (*p* < 0.05), # indicates difference between the oe-ADCY3 + RMI 12330A group and the oe-ADCY3 + DMSO group (*p* < 0.05). **(D,E)** Expression levels of ADCY3, CREB, pCREB, cAMP, and PKA in mice as detected by Western Blot and ELISA, *n* = 6, *indicates difference between the oe-ADCY3 group and the oe-NC group (*p* < 0.05), ns indicates no difference between the oe-ADCY3 + RMI 12330A group and the oe-ADCY3 + DMSO group (*p* > 0.05). **(F)** Detection of sIPSC using electrophysiological techniques, scale bar, 100 ms and 1,000 pA, *n* = 20 neurons, bar graph (right) represents frequency and amplitude. **(G)** Detection of eIPSC using electrophysiological techniques, scale bar, 2 s and 20 pA, *n* = 20 neurons, bar graph (right) represents amplitude. **(H)** Measurement of the relative intensity changes of calcium ions in different groups of mice neurons using calcium imaging techniques (scale bar = 25 μm), *n* = 10 cells, *indicates difference between the oe-ADCY3 group and the oe-NC group (*p* < 0.05), # indicates difference between the oe-ADCY3 + RMI 12330A group and the oe-ADCY3 + DMSO group (*p* < 0.05).

Behavioral observations were conducted on mice, including evaluation of mouse performance using the Racine scoring system, electroencephalogram (EEG) detection, and recording of epilepsy frequencies. Racine scoring results showed that compared to the oe-NC group, the epilepsy score decreased in the oe-ADCY3 group; compared to the oe-ADCY3 + DMSO group, the epilepsy score increased in the oe-ADCY3 + RMI 12330A group (Figure 6A). EEG results showed that compared with the oe-NC group, the amplitude of epileptic EEG activity was reduced in the oe-ADCY3 group; compared with the oe-ADCY3 + DMSO group, the amplitude of epileptic EEG activity was increased in the oe-ADCY3 + RMI 12330A group (Figure 6B). Statistical analysis of the frequency of epileptic seizures revealed a significant reduction in seizure frequency in the oe-ADCY3 group compared to the oe-NC group. Conversely, when comparing mice in the oe-ADCY3 + DMSO group (overexpression lentivirus ADCY3 group with intraperitoneal injection of varying doses of DMSO) to those in the oe-ADCY3 + RMI 12330A group (overexpression lentivirus ADCY3 group with intraperitoneal injection of varying doses of cAMP inhibitor, RMI 12330A), there was an increase in seizure frequency in the oe-ADCY3 + RMI 12330A group (Figure 6C).

To explore the molecular mechanism of treating epileptic epilepsy by activating the cAMP signaling pathway through ADCY3, we utilized the Western blot analysis method to examine the protein expression levels of ADCY3, cAMP-responsive element binding protein (CREB), and phosphorylated CREB (pCREB). Additionally, we employed the ELISA analysis method to measure the expression levels of cAMP and protein kinase A (PKA). The results showed that compared with the oe-NC group, the expression levels of cAMP, PKA, ADCY3, CREB, and pCREB were upregulated in the oe-ADCY3 group. Compared with the oe-ADCY3 + DMSO group, the expression levels of cAMP, PKA, CREB, and pCREB were downregulated in the oe-ADCY3 + RMI 12330A group, while the expression level of ADCY3 showed no difference (Figures 6D,E). These results indicate that overexpression of ADCY3 affects the production of cAMP.

Furthermore, we investigated whether activating the ADCY3/cAMP signaling pathway can enhance neuronal inhibition and improve epilepsy onset in epilepsy. We analyzed the inhibitory properties of neurons using electrophysiological techniques, as described in the methods section. Electrophysiological techniques recorded spontaneous inhibitory postsynaptic currents (sIPSCs) and evoked inhibitory postsynaptic currents (PSCs). UPSC results showed that compared with the oe-NC group, the frequency and amplitude increased in the oe-ADCY3 group, and the inhibitory effect of neurons was enhanced. Compared with the oe-ADCY3 + DMSO group, the amplitude decreased, and the inhibitory effect of neurons was weakened in the oe-ADCY3 + RMI 12330A group (Figure 6F). The UPSC results showed that compared to the oe-NC group, the frequency and amplitude increased in the oe-ADCY3 group, and the inhibitory effect of the neurons was enhanced. Compared to the oe-ADCY3 + DMSO group, the amplitude decreased, and the inhibitory effect of the neurons was weakened in the oe-ADCY3 + RMI 12330A group (Figure 6G).

Calcium ions are one of the critical mediators for signal transmission in neurons. After the cell membrane is stimulated, calcium ion channels on the membrane can either open or close, resulting in the influx of calcium ions and an increase in calcium ion concentration, which has a particular impact on the excitability of neurons (Ross, 1989). Hence, we utilized calcium imaging analysis to examine the trends in calcium ion concentration changes in neurons, thereby investigating the impact of ADCY3 activation on neuronal excitability via the cAMP signaling pathway. The results indicate that compared to the oe-NC group, the oe-ADCY3 group exhibited a decreasing trend in calcium ion concentration. Additionally, when comparing the oe-ADCY3 + DMSO group with the oe-ADCY3 + RMI 12330A group, there was an increasing trend in the relative intensity of calcium fluorescence signals in the latter (Figure 6H).

In conclusion, modulating the activation of ADCY3 can enhance inhibitory synaptic transmission, increase neuronal inhibition, and thereby improve epilepsy onset in epilepsy.

### 3.4 A ketogenic diet regulates ADCY3 to activate the cAMP signaling pathway and improve epileptic epilepsy

To investigate the regulatory effects of the ketogenic diet on ADCY3-mediated activation of the cAMP signaling pathway and its impact on epileptic epilepsy, we further validated the influence of the ketogenic diet on the cAMP signaling pathway and epilepsy occurrence. We established a mouse model of epilepsy and performed experimental verification using a cAMP inhibitor. The experiment is divided into the KD group and the KD + RMI 12330A group.

**Figure 7:**
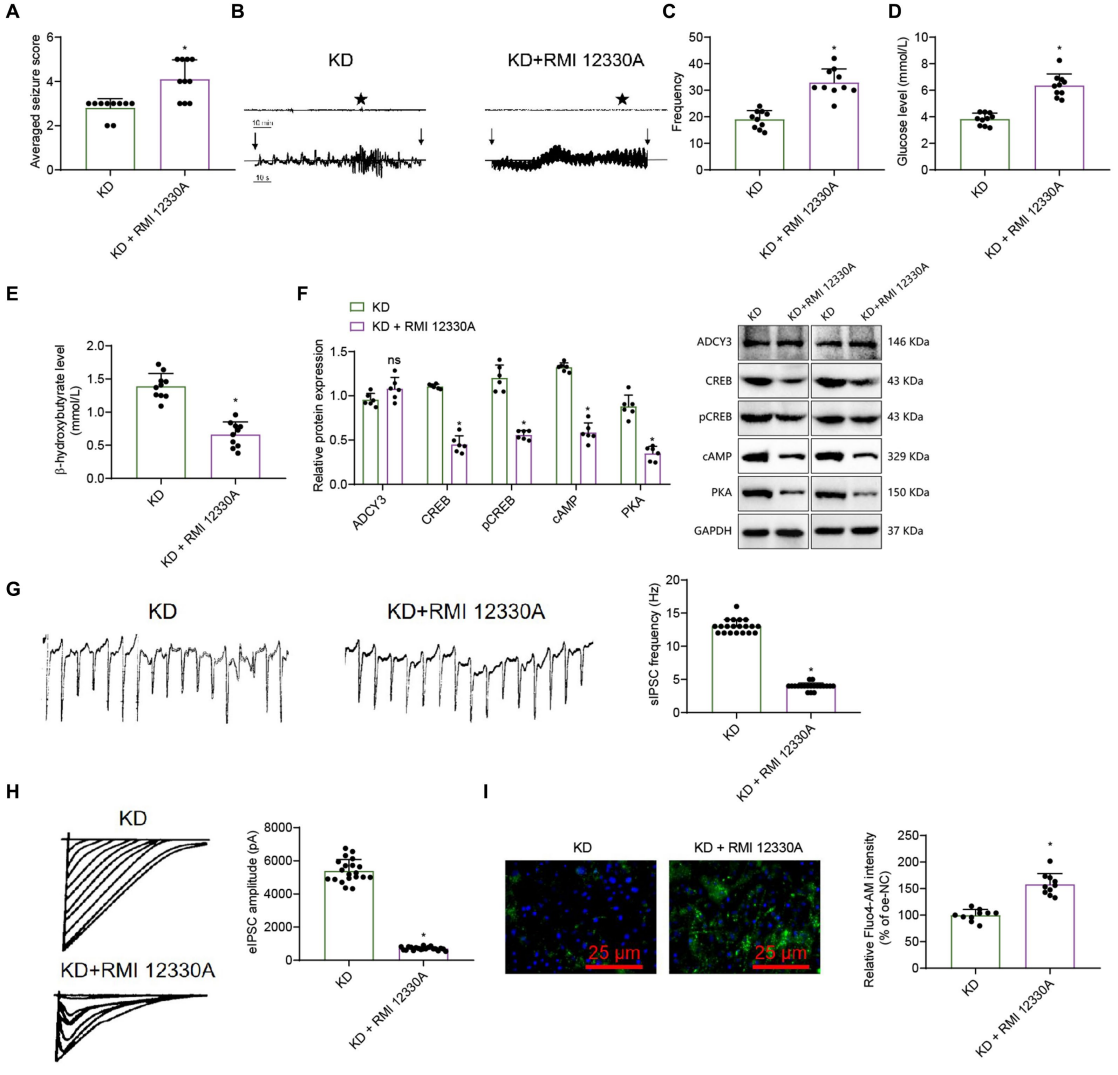
The ketogenic diet regulates the cAMP signaling pathway and influences epileptic seizure. **(A)** Behavioral observation in mice (Racine score), *n* = 10, * indicates difference between the KD + RMI 12330A group and the KD group (*p* < 0.05). **(B)** Analysis of mouse EEG, and vertical arrows indicate the beginning and end of seizures in mice. **(C)** Frequency of epileptic epilepsy, *n* = 10, * indicates difference between the KD + RMI 12330A group and the KD group (*p* < 0.05). **(D,E)** Levels of β-hydroxybutyrate and Glucose, *n* = 10, * indicates difference between the KD + RMI 12330A group and the KD group (*p* < 0.05). **(F)** Western Blot analysis of ADCY3, CREB, pCREB, cAMP, and PKA expression levels in mice, *n* = 6, * indicates difference between the KD + RMI 12330A group and the KD group (*p* < 0.05), ns indicates no difference between the KD + RMI 12330A group and the KD group (*p* > 0.05). **(G)** Detection of sIPSC using electrophysiological techniques, bar graph (right) represents frequency and amplitude, scale bar, 100 ms and 1,000 pA, *n* = 20 neurons. **(H)** Detection of eIPSC using electrophysiological techniques, bar graph (right) represents amplitude, scale bar, 2 s and 20 pA, *n* = 20 neurons. **(I)** Measurement of the relative intensity in calcium ion concentrations among the different groups of mice’s neurons using calcium imaging techniques (scale bar = 25 μm), *n* = 10 cells, * indicates difference between the KD + RMI 12330A group and the KD group (*p* < 0.05).

We conducted behavioral observations on mice, and according to the Racine scoring results, the epilepsy score of the KD + RMI 12330A group was higher compared to the KD group (Figure 7A). The electroencephalogram results showed that compared with the KD group, the amplitude of epileptic brain electrical activity in the KD + RMI 12330A group increased (Figure 7B). Analysis of epilepsy frequency statistics showed that compared to the KD group, the KD + RMI 12330A group had increased epilepsy frequency (Figure 7C).

β-hydroxybutyrate and glucose levels reflect the body’s fat metabolism status. Compared to the KD group, the KD + RMI 12330A group showed a decrease in β-hydroxybutyrate concentration and an increase in blood glucose levels (Figures 7D,E), indicating that the ketogenic diet can promote fatty acid metabolism and improve epilepsy disorders.

We used the Western blot analysis to detect cAMP, PKA, ADCY3, CREB, and pCREB protein expression levels. The results showed that compared to the KD group, the expression levels of cAMP, PKA, CREB, and pCREB in the KD + RMI 12330A group were down-regulated, while the expression level of ADCY3 showed no difference (Figure 7F).

We used electrophysiological techniques to analyze spontaneous inhibitory postsynaptic currents (sIPSCs) and evoked inhibitory postsynaptic currents (PSCs). The sIPSC results revealed that, compared to the KD group, the KD + RMI 12330A group exhibited a decreased frequency and significantly reduced amplitude, indicating a marked decrease in neuronal inhibitory function (Figure 7G). The UPSC results showed that compared to the KD group, the amplitude of the KD + RMI 12330A group decreased, and the inhibitory nature of the neurons was weakened (Figure 7H).

We employed calcium imaging analysis to detect the trends in the relative intensity changes of calcium fluorescence signals in neurons. The results revealed that compared to the KD group, the KD + RMI 12330A group exhibited an increasing trend in the relative intensity of calcium fluorescence signals, indicating a decrease in neuronal excitability (Figure 7I).

In summary, by regulating ADCY3 to activate the cAMP signaling pathway and increasing inhibitory synaptic transmission, the ketogenic diet improves epilepsy activity in epilepsy.

### 3.5 The impact of a classical ketogenic diet on patients with epileptic epilepsy

In order to evaluate the efficacy of the ketogenic diet in patients with epilepsy, we conducted a study involving 60 individuals diagnosed with epilepsy. These participants were randomly assigned to either a control group or an observation group, with each group consisting of 30 individuals. Comparison of the general characteristics between the two groups revealed no significant differences (*p* > 0.05), indicating their comparability (Supplementary Table S6). The Hospital’s Medical Ethics Committee has approved this study.

**Figure 8:**
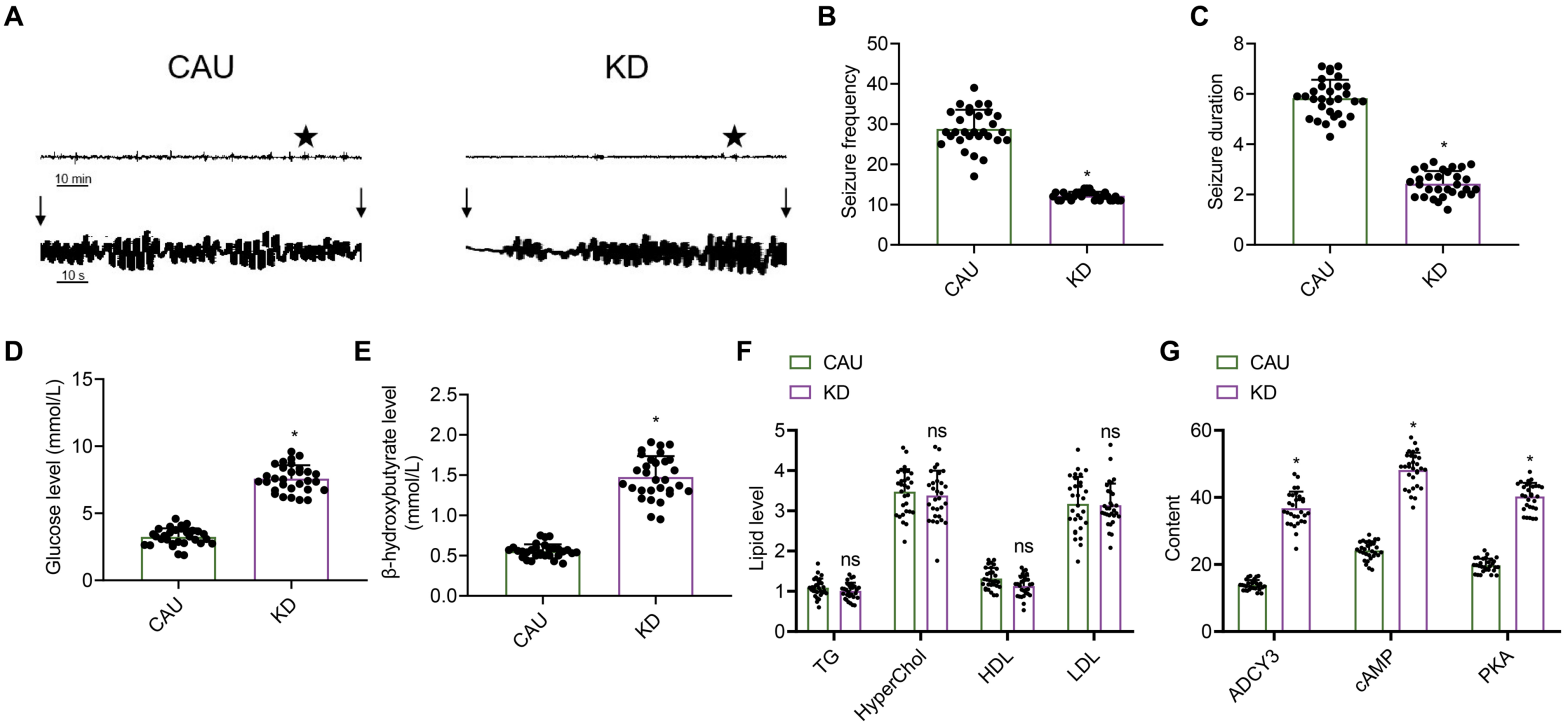
Effect of the classical ketogenic diet on epilepsy onset in patients with epilepsy. CAU, conventional epilepsy diet group; KD, classical ketogenic diet group. **(A)** EEG of patients with epilepsy, *n* = 30, and the vertical arrow indicates the beginning and end of epileptic seizures in mice. **(B)** Frequency of epilepsy in patients with epilepsy, *n* = 30, * indicates the comparison between the two groups, *p* < 0.05. **(C)** Duration of epilepsy in patients with epilepsy, *n* = 50, * indicates the comparison between the two groups, *p* < 0.05. **(D)** Glucose concentration levels, *n* = 50, * indicates the comparison between the two groups, *p* < 0.05. **(E)** β-hydroxybutyrate levels, *n* = 50, * indicates the comparison between the two groups, *p* < 0.05. **(F)** Influence of KD on blood lipid levels in patients with epilepsy, *n* = 30, TG (triglycerides), HyperChol (cholesterol), HDL (high-density lipoprotein), LDL (low-density lipoprotein), ns indicates the comparison between the two groups, *p* > 0.05. **(G)** Expression of ADCY3, cAMP, and PKA in the blood of patients with epilepsy detected by ELISA, *n* = 30, * indicates the comparison between the two groups, *p* < 0.05.

The electroencephalogram (EEG) results of patients with epilepsy showed that compared to the control group, the ketogenic diet group had a decreased amplitude of EEG activity (Figure 8A). Compared with the control group, the ketogenic diet group showed a reduction in the frequency and duration of epileptic epilepsy (Figures 8B,C), indicating that the ketogenic diet can decrease the frequency and duration of epilepsy in epileptic patients, thereby improving their epilepsy condition. Compared with the control group, the ketogenic diet group showed a decrease in glucose levels and an increase in β-hydroxybutyrate levels, indicating that the ketogenic diet can elevate ketone body levels in patients and promote fatty acid metabolism, thereby improving epilepsy in epilepsy (Figures 8D,E). Blood lipid analysis results showed no differences in cholesterol, triglycerides, high-density lipoprotein, and low-density lipoprotein between the ketogenic diet group and the control group (Figure 8F), indicating that the ketogenic diet does not cause harm to patients. In addition, we also assessed the expression levels of ADCY3, cAMP, and PKA in the blood of patients with epilepsy using ELISA. The results showed that in the classical ketogenic diet group, ADCY3, cAMP, and PKA expression levels were upregulated compared to the control group (Figure 8G). It indicates that the classical ketogenic diet regulates the ADCY3-initiated cAMP signaling pathway by promoting fatty acid metabolism, inhibiting neuronal activity and further alleviating patients’ epileptic epilepsy.

## 4 Discussion

In this study, we delved into the molecular mechanisms by which the ketogenic diet regulates fatty acid metabolism, activates ADCY3 to initiate the cAMP signaling pathway, enhances neuronal inhibition, and treats epileptic seizures. Through high-throughput transcriptome sequencing and *in vivo* experiments in mice, we systematically elucidated how the ketogenic diet increases neuronal inhibition and improves seizure control by promoting fatty acid metabolism to regulate ADCY3 activation of the cAMP signaling pathway. The findings of this study align with previous clinical and experimental research, confirming the effectiveness of the ketogenic diet in epilepsy treatment (Wells et al., 2020; Kong et al., 2021).

Our meta-analysis revealed that the efficacy of the ketogenic diet in treating epilepsy surpasses that of conventional diets, with the classical ketogenic diet demonstrating superior improvement in seizure activity compared to other variants. These results provide robust evidence for clinicians to consider the ketogenic diet as a non-pharmacological treatment option for epilepsy patients, guiding clinical practices coherently with previous research. Both traditional and network meta-analyses support the superior efficacy of the classical ketogenic diet in ameliorating seizure activity compared to other variants (Wang et al., 2022), endorsing the ketogenic diet as a viable non-pharmacological therapeutic approach (Yuan et al., 2020; Sun et al., 2021; Ruan et al., 2022).

The meta-analysis revealed that the classical ketogenic diet may improve epileptic seizures by modulating the expression of VAV2, CALM3, ADCY3, CACNA1S, PRKACA, and GRIN2B, thereby regulating the cAMP signaling pathway to inhibit neuronal activity (Figures 1–4). Subsequently, the expression of six key genes was examined in the hippocampal tissue of epileptic mice (Figure 5). Based on the literature, ADCY3, identified as a significant adenylyl cyclase, when activated, elevates intracellular cAMP levels and modulates various cellular functions (Son et al., 2022; Gárriz et al., 2023). Consequently, ADCY3 is viewed as a potential therapeutic target for epilepsy treatment. Furthermore, a mouse model was successfully established to validate the findings. It was discovered that modulating ADCY3 to initiate the cAMP signaling pathway could enhance inhibitory synaptic transmission, increasing neuronal inhibition and ameliorating epileptic seizures (Figure 6). This research highlights the critical role of ADCY3 in the ketogenic diet and lays the groundwork for further investigations into its mechanism of action. This finding aligns with existing literature, emphasizing the vital regulatory role of the cAMP signaling pathway in treating epileptic seizures with a ketogenic diet. cAMP influences neuronal function by activating protein kinase A (PKA) and regulating the phosphorylation of the transcription factor CREB (Grigsby et al., 2020). Additionally, delving deeper into the specific regulatory mechanisms of ADCY3 within the cAMP signaling pathway and exploring other potential molecular mechanisms warrants further investigation (Ding et al., 2021).

Subsequently, we validated that the ketogenic diet can improve epileptic seizures by modulating ADCY3 to activate the cAMP signaling pathway (Figure 7). This discovery holds significant importance for gaining a deeper understanding of the molecular mechanisms underlying epileptic seizures and exploring novel therapeutic approaches. This conclusion is consistent with research findings from relevant literature, indicating that through cAMP pathway activation, the ketogenic diet can enhance neuronal inhibition, reducing the likelihood of epileptic seizures (Luo et al., 2022).

Lastly, to ascertain the efficacy of the ketogenic diet in human epilepsy patients, we collected data from 60 epilepsy patients. The results demonstrated that the classical ketogenic diet regulates ADCY3 to initiate the cAMP signaling pathway by promoting fatty acid metabolism, thereby inhibiting neuronal activity and further alleviating the occurrence of seizures in patients (Figure 8). As a non-pharmacological treatment approach, the ketogenic diet exhibits fewer side effects and dependencies, making it a promising option for certain refractory epilepsy patients (Paoli et al., 2020). Future clinical studies could further evaluate the application effectiveness of the ketogenic diet in various types of epilepsy patients and explore its combined use with medication or other treatment modalities (Norwitz et al., 2020). Additionally, long-term follow-up observations can assess the impact of the ketogenic diet on the recurrence rate of epileptic seizures and long-term prognosis, providing stronger evidence for clinical practice (Desli et al., 2022).

**Figure 9:**
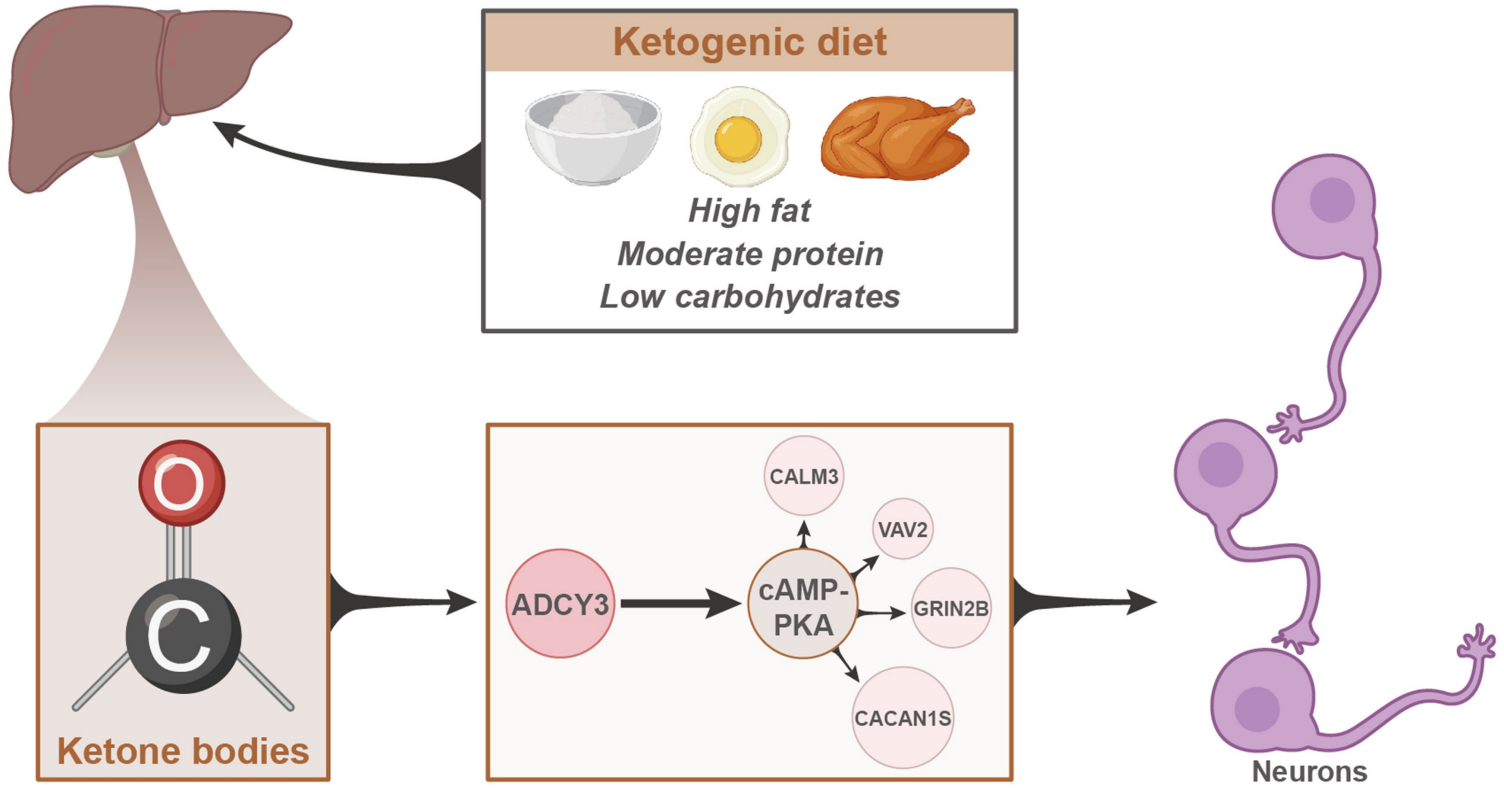
Schematic representation of the molecular mechanisms underlying the therapeutic effects of the classical ketogenic diet in epilepsy.

In conclusion, we can preliminarily deduce the following outcomes: the classical ketogenic diet enhances neuronal inhibition by promoting fatty acid release, thereby regulating ADCY3 to activate the cAMP signaling pathway, leading to the amelioration of epileptic seizures (Figure 9). This study provides significant scientific and clinical value by delving into the mechanisms of the ketogenic diet in treating epileptic seizures.

However, this study has certain limitations. Although we employed mouse models and transcriptome sequencing for validation, there are inherent methodological constraints. Future research could utilize alternative models such as large animal models or *in vitro* cell models to better mimic the pathological processes of human epilepsy. Moreover, integrating other technologies like single-cell transcriptome sequencing and proteomics analysis could further elucidate the regulatory mechanisms of the ketogenic diet on neuronal inhibition. While this study offers initial insights into the molecular mechanisms of the ketogenic diet in epilepsy treatment, there are numerous aspects that require further exploration, such as investigating the diet’s regulatory effects on other epilepsy-related genes and elucidating the specific functions of these genes during seizure onset (Sourbron et al., 2020; Wells et al., 2020; Dashti et al., 2021).

The systemic effects of intraperitoneal administration on *in vivo* validation were not extensively investigated in this study, introducing certain limitations that need exploration in future research. Predominantly based on animal experiments and transcriptome sequencing analyses, large-scale clinical trials have not yet been conducted for verification. Therefore, it is imperative to proceed with further human clinical studies to validate the efficacy and safety of the ketogenic diet in epilepsy patients. Additionally, the limited selection of ketogenic diet types in this study, including the classical ketogenic diet, MAD, LGIT, and MCT, leaves other variants unexplored. Hence, further research is essential to investigate the mechanisms of action of other types of ketogenic diets in epilepsy treatment. Furthermore, Fluo-4 AM is not the optimal dye for measuring the ratio metric of absolute calcium concentrations between independent samples. In future studies, we will consider using calcium indicators like Fura Red AM for ratio metric calcium measurement to enhance the accuracy of our calcium measurements.

Despite its limitations, the results of this study offer promising prospects. Firstly, further research could broaden the spectrum of ketogenic diet types, comparing the efficacy of different types in epilepsy treatment to identify more personalized dietary strategies. Secondly, exploring the therapeutic effects of the ketogenic diet on other neurological disorders such as Parkinson’s and Alzheimer’s diseases is warranted. Investigating the common mechanisms of action of the ketogenic diet on neurological diseases could extend its applications across various disease domains. Additionally, combining the ketogenic diet with other therapies like drug treatments and neuroregulation techniques could enhance the effectiveness of epilepsy treatment.

In essence, our study provides a profound understanding of the application of the ketogenic diet in epilepsy treatment, offering a non-pharmacological treatment option for epilepsy patients. Future research will refine the application strategies of the ketogenic diet, exploring its potential value in other neurological disorders, fostering new breakthroughs in both neuroscience research and clinical practice.

## Data availability statement

The original contributions presented in the study are included in the article/Supplementary material, further inquiries can be directed to the corresponding authors.

## Ethics statement

All mice were handled strictly with the ethical requirements for experimental animals and obtained approval from the Animal Ethics Committee for Experimentation (IACUC FJMU 2023-0100). The studies were conducted in accordance with the local legislation and institutional requirements. The participants provided their written informed consent to participate in this study. This study has been approved by the Clinical Ethics Committee of Fujian Medical University Union Hospital, and written informed consent has been obtained from the patients and their families, strictly adhering to the Helsinki Declaration (2022YF018-01). The study was conducted in accordance with the local legislation and institutional requirements.

## Author contributions

ML: Conceptualization, Formal analysis, Methodology, Writing – original draft, Writing – review & editing. JG: Conceptualization, Methodology, Supervision, Validation, Writing – original draft, Writing – review & editing. LW: Conceptualization, Methodology, Supervision, Validation, Writing – review & editing. XL: Data curation, Resources, Validation, Writing – review & editing. YZ: Data curation, Resources, Validation, Writing – review & editing. WL: Formal analysis, Investigation, Supervision, Writing – review & editing. HH: Formal analysis, Investigation, Project administration, Writing – original draft, Writing – review & editing. CZ: Formal analysis, Investigation, Project administration, Writing – original draft, Writing – review & editing.

## Supplementary material

SUPPLEMENTARY FIGURE S1 Literature screening process.

SUPPLEMENTARY FIGURE S2 Cochrane bias risk assessment. Green represents low risk, red represents high risk, and yellow represents potential high bias risk.

SUPPLEMENTARY FIGURE S3 Network diagram comparing the efficacy of different types of ketogenic diets. **(A)** ≥50% reduction in epilepsy at 3 months; **(B)** ≥90% reduction in epilepsy at 3 months; **(C)** ≥50% reduction in epilepsy at 6 months; **(D)** ≥90% reduction in epilepsy at 6 months. The lines connecting the nodes indicate their correlations, with more lines indicating higher correlations.

SUPPLEMENTARY FIGURE S4 Forest plot of meta-analysis comparing the efficacy of different types of ketogenic diets. **(A)** ≥50% reduction in epilepsy at 3 months; **(B)** ≥90% reduction in epilepsy at 3 months; **(C)** ≥50% reduction in epilepsy at 6 months; **(D)** ≥90% reduction in epilepsy at 6 months.
