# Supplementary figures and images for: ADCY3: the pivotal gene in classical ketogenic diet for the treatment of epilepsy
Source: Front Cell Neurosci. 2024 May 22;18:1305867. doi: 10.3389/fncel.2024.1305867 (PMC11150708; doi:10.3389/fncel.2024.1305867)

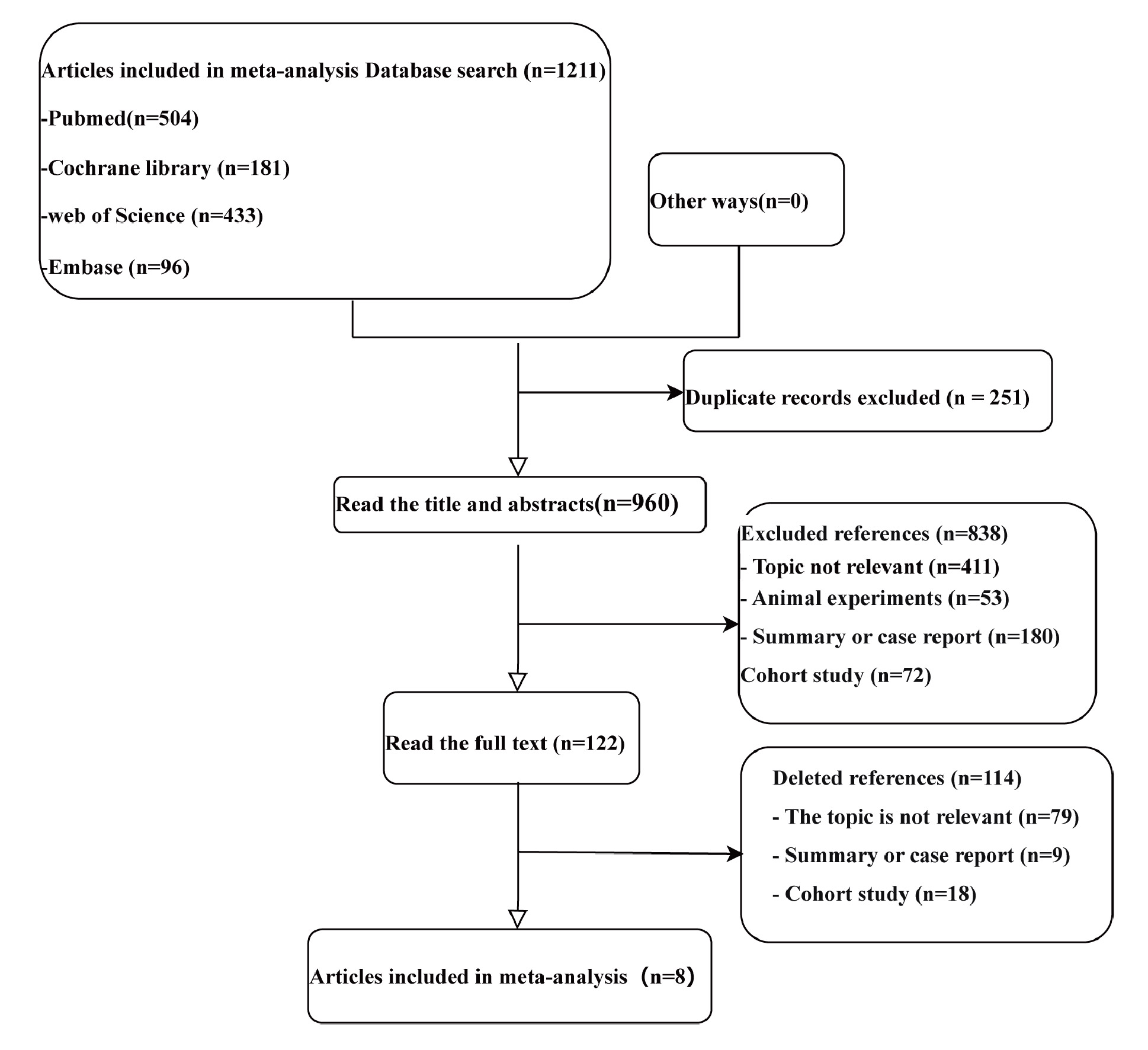

Supplement: SUPPLEMENTARY FIGURE S1 — Literature screening process. [file Image_1.JPEG]

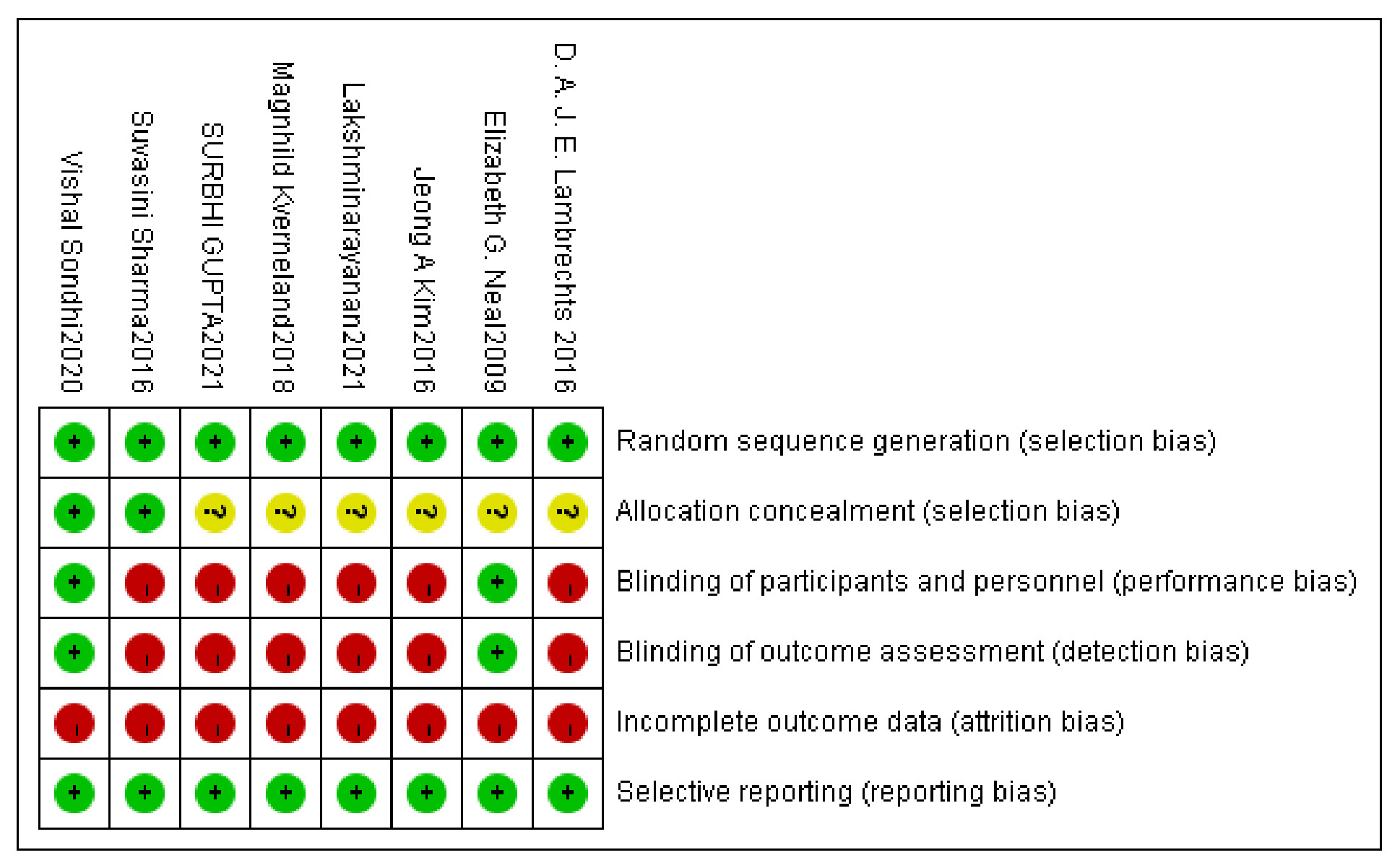

Supplement: SUPPLEMENTARY FIGURE S2 — Cochrane bias risk assessment. Green represents low risk, red represents high risk, and yellow represents potential high bias risk. [file Image_2.JPEG]

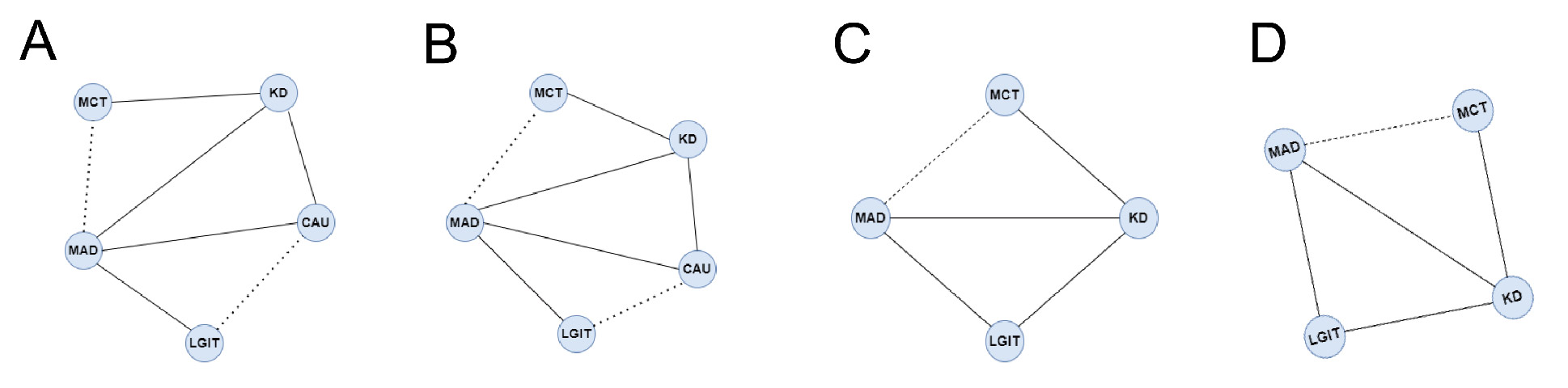

Supplement: SUPPLEMENTARY FIGURE S3 — Network diagram comparing the efficacy of different types of ketogenic diets. (A) ≥50% reduction in epilepsy at 3 months; (B) ≥90% reduction in epilepsy at 3 months; (C) ≥50% reduction in epilepsy at 6 months; (D) ≥90% reduction in epilepsy at 6 months. The lines connecting the nodes indicate their correlations, with more lines indicating higher correlations. [file Image_3.JPEG]

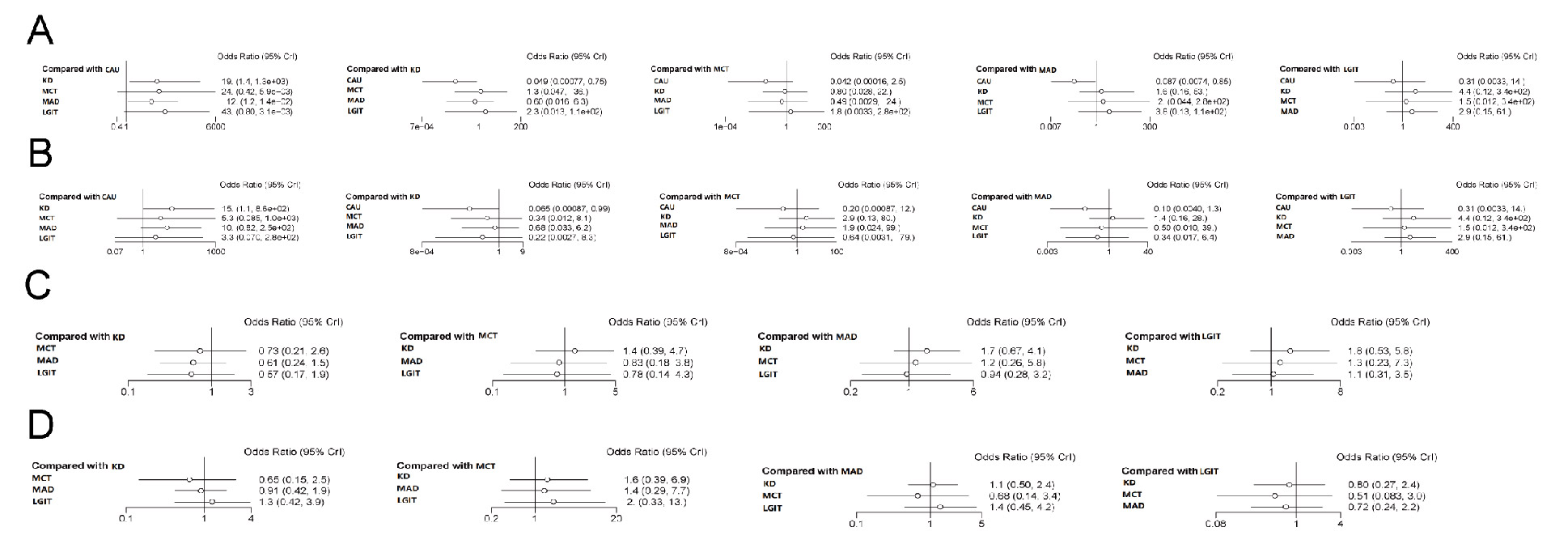

Supplement: SUPPLEMENTARY FIGURE S4 — Forest plot of meta-analysis comparing the efficacy of different types of ketogenic diets. (A) ≥50% reduction in epilepsy at 3 months; (B) ≥90% reduction in epilepsy at 3 months; (C) ≥50% reduction in epilepsy at 6 months; (D) ≥90% reduction in epilepsy at 6 months. [file Image_4.JPEG]
