# Supplementary material for: ADCY3: the pivotal gene in classical ketogenic diet for the treatment of epilepsy
Source: Front Cell Neurosci. 2024 May 22;18:1305867. doi: 10.3389/fncel.2024.1305867 (PMC11150708; doi:10.3389/fncel.2024.1305867)
Supplement: Supplementary file 5 [file Table_1.DOCX]

**Table S1. RT-qPCR Primer sequence(mouse)**

| Gene | Primer sequence |
| --- | --- |
| VAV2(mouse) | Forward:5’-GCCCATGAAAATGGGCATGA-3’ |
|  | Reverse:5’-GCTCGCAGAAAGCTGTGATG-3’ |
| ADCY3 (mouse) | Forward:5’-AGGTCAGCCACATGGTGAAG-3’ |
|  | Reverse:5’-TCAGTGCCATTGAGCCCAG-3’ |
| CACNA1S(mouse) | Forward:5’-AGGGGCAAAAGCAGAGCATC-3’ |
|  | Reverse:5’-GCCTGTTGCCATGACGAAGT-3’ |
| CALM3(mouse) | Forward:5’-CCCTGTGACTCTGTGTGCAA-3’ |
|  | Reverse:5’-CGTCCACTCTATGCAGTCCC-3’ |
| PRKACA(mouse) | Forward:5’-CCCCTACCCTGCACCCATTA-3’ |
|  | Reverse:5’-ACATCGTTGGAGCTGGAAGC-3’ |
| GRIN2B(mouse) | Forward:5’-GAGCACGTGGACTTGACTGA-3’ |
|  | Reverse:5’-CCCCAGACCTATCCTCCCAA-3’ |
| GAPDH(mouse) | Forward:5’-GCCTCCTCCAATTCAACCCT-3’ |
|  | Reverse:5’-CTCGTGGTTCACACCCATCA-3’ |
