# Supplementary material for: ADCY3: the pivotal gene in classical ketogenic diet for the treatment of epilepsy
Source: Front Cell Neurosci. 2024 May 22;18:1305867. doi: 10.3389/fncel.2024.1305867 (PMC11150708; doi:10.3389/fncel.2024.1305867)
Supplement: Supplementary file 6 [file Table_2.DOCX]

**Table S2. Study characteristics**

| Number/Author/Year | Country | Research type | Age/years | Total sample size/cases | Grouping of intervention measures/examples | Follow-up time (months) | Observation indicators |
| --- | --- | --- | --- | --- | --- | --- | --- |
| ①D. A. J. E. Lambrechts1/2016(PMID: 27027847) | Netherlands | RCT | 1-18 | 48 | KD/CAU  26/22 | 12 | ab |
| ②Magnhild Kverneland/2018  (PMID: 29901816) | Norway | RCT | ＞16 | 56 | MAD/CAU  24/32 | 3 | a |
| ③Lakshminarayanan/2021  (PMID: 33582533) | United States | RCT | 2-15 | 40 | LGIT/CAU  20/20 | 3 | a |
| ④Suvasini Sharma/2016(PMID: 27603509) | India | RCT | 2-14 | 81 | MAD/CAU  41/40 | 3 | abe |
| ⑤Elizabeth G. Neal/2009  (PMID: 19054400) | United Kingdom | RCT | 2-16 | 145 | KD/MCT  73/72 | 6 | abcd |
| ⑥Vishal Sondhi/2020(PMID: 32761191 ) | India | RCT | 1-15 | 158 | KD/MAD  /LGIT  52/52/54 | 6 | cd |
| ⑦Jeong A Kim/2016  (PMID: 26662710) | Korea | RCT | 1-18 | 104 | KD/MAD  51/53 | 3 | abcde |
| ⑧SURBHI GUPTA/2021  (PMID: 27603509) | India | RCT | 0.5-14 | 60 | MAD/LGIT  30/30 | 3 | abe |

Note: CAU, conventional diet; KD, classic ketogenic diet group; MCT, medium-chain triglyceride ketogenic diet; MAD, modified Atkins diet; LGIT, low glycemic index ketogenic diet. a = reduction in seizures by ≥50% for 3 months, b = reduction in seizures by ≥90% for 3 months, c = reduction in seizures by ≥50% for 6 months (24 weeks), d = reduction in seizures by ≥90% for 6 months (24 weeks), e = seizure free (no seizures at all).
