# Supplementary material for: ADCY3: the pivotal gene in classical ketogenic diet for the treatment of epilepsy
Source: Front Cell Neurosci. 2024 May 22;18:1305867. doi: 10.3389/fncel.2024.1305867 (PMC11150708; doi:10.3389/fncel.2024.1305867)
Supplement: Supplementary file 7 [file Table_3.DOCX]

**Table S3. Risk of bias summary(Jadad)**

| Include in the study | Random method | Randomization concealment | Blind law | Exit and loss Visit description | Continuity of medical information | Follow-up time (months) | Follow-up time (months) |
| --- | --- | --- | --- | --- | --- | --- | --- |
| D. A. J. E. Lambrechts1 | Appropriate | Unclear | Inappropriate | Have | Is | 12 | 4 |
| Magnhild Kverneland | Appropriate | Unclear | Inappropriate | Have | Is | 3 | 4 |
| Lakshminarayanan | Appropriate | Unclear | Inappropriate | Have | Is | 3 | 4 |
| Suvasini Sharma | Appropriate | Appropriate | Inappropriate | Have | Is | 3 | 5 |
| Elizabeth G. Neal | Appropriate | Unclear | Appropriate | Have | Is | 6 | 6 |
| Vishal Sondhi | Appropriate | Appropriate | Appropriate | Have | Is | 6 | 7 |
| Jeong A Kim | Appropriate | Unclear | Inappropriate | Have | Is | 3 | 4 |
| SURBHI GUPTA | Appropriate | Unclear | Inappropriate | Have | Is | 3 | 4 |
