# Supplementary material for: ADCY3: the pivotal gene in classical ketogenic diet for the treatment of epilepsy
Source: Front Cell Neurosci. 2024 May 22;18:1305867. doi: 10.3389/fncel.2024.1305867 (PMC11150708; doi:10.3389/fncel.2024.1305867)
Supplement: Supplementary file 8 [file Table_4.DOCX]

**Table S4. Seizure reduction ≥50% [OR(95%CI)]**

| Clinical effectiveness ratio (OR value) between various intervention measures | | CAU | | KD | | MCT | | MAD | | LGIT | |
| --- | --- | --- | --- | --- | --- | --- | --- | --- | --- | --- | --- |
|  |  | 3 months | 6months | 3months | 6months | 3months | 6months | 3months | 6months | 3months | 6months |
| CAU | 3months | - | - | 0.049 (0.00077, 0.75) | - | 0.042 (0.00016, 2.5) | - | 0.087 (0.0074, 0.85) | - | 0.023 (0.00032, 1.4) | - |
|  | 6 months | - | - | - | - | - | - | - | - | - | - |
| KD | 3months | 19. (1.4, 1300) | - | - | - | 0.80 (0.028, 22.) | - | 1.6 (0.16, 53.) | - | 0.44 (0.0088, 76.) | - |
|  | 6months | - | 16. (1.1, 1100) | - | - | - | 1.4 (0.39, 4.7) | - | 1.7 (0.67, 4.1) | - | 1.8 (0.53, 5.8) |
| MCT | 3months | 24. (0.42,5900) | - | 1.3 (0.047, 36.) | - | - | - | 2. (0.044, 280) | - | 0.55 (0.0037, 280) | - |
|  | 6months | - | 5.4 (0.083, 1300) | - | 0.73 (0.21, 2.6) | - | - | - | 1.2 (0.26, 5.8) | - | 1.3 (0.23, 7.3) |
| MAD | 3months | 12. (1.2, 140) | - | 0.60 (0.016, 6.3) | - | 0.49 (0.0029, 24.) | - | - | - | 0.26 (0.0090, 7.7) | - |
|  | 6months | - | 10. (0.82, 290) | - | 0.61 (0.24, 1.5) | - | 0.83 (0.18, 3.8) | - | - | - | 1.1 (0.31, 3.5) |
| LGIT | 3months | 43. (0.80, 3100) | - | 2.3 (0.013, 110) | - | 1.8 (0.0033, 280) | - | 3.8 (0.13, 110) | - | - | - |
|  | 6months | - | 3.5 (0.072, 350) | - | 0.57 (0.17, 1.9) | - | 0.78 (0.14, 4.3) | - | 0.94 (0.28, 3.2) | - | - |
