# Supplementary material for: ADCY3: the pivotal gene in classical ketogenic diet for the treatment of epilepsy
Source: Front Cell Neurosci. 2024 May 22;18:1305867. doi: 10.3389/fncel.2024.1305867 (PMC11150708; doi:10.3389/fncel.2024.1305867)
Supplement: Supplementary file 9 [file Table_5.DOCX]

**Table S5. Seizure reduction ≥90% [OR(95%CI)]**

| Clinical effectiveness ratio (OR value) between various intervention measures | | CAU | | KD | | MCT | | MAD | | LGIT | |
| --- | --- | --- | --- | --- | --- | --- | --- | --- | --- | --- | --- |
|  |  | 3months | 6months | 3months | 6months | 3months | 6months | 3months | 6months | 3months | 6months |
| CAU | 3months | - | - | 0.065 (0.00087, 0.99) | - | 0.20 (0.00087, 12.) | - | 0.10 (0.0040, 1.3) | - | 0.31 (0.0033, 14.) | - |
|  | 6months | - | - | - | - | - | - | - | - | - | - |
| KD | 3months | 15. (1.1, 860) | - | - | - | 2.9 (0.13, 80.) | - | 1.4 (0.16, 28.) | - | 4.4 (0.12, 340) | - |
|  | 6months | - | - | - | - | - | 1.6 (0.39, 6.9) |  | - | - | 0.80 (0.27, 2.4) |
| MCT | 3months | 5.3 (0.085, 1000) | - | 0.34 (0.012, 8.1) | - | - | - | 0.50 (0.010, 39.) | - | 1.5 (0.012, 340) | - |
|  | 6months | - | - | - | 0.65 (0.15, 2.5) | - | - | - | - | - | 0.51 (0.083, 3.0) |
| MAD | 3months | 10. (0.82, 250) | - | 0.68 (0.033, 6.2) | - | 1.9 (0.024, 99.) | - | - | - | 2.9 (0.15, 61.) | - |
|  | 6months | - | - | - | 0.91 (0.42, 1.9) | - | 1.4 (0.29, 7.7) | - | - | - | 0.72 (0.24, 2.2) |
| LGIT | 3months | 3.3 (0.070,280) | - | 0.22 (0.0027, 8.3) | - | 0.64 (0.0031, 79.) | - | 0.34 (0.017, 6.4) | - | - | - |
|  | 6months | - | - | - | 1.3 (0.42, 3.9) | - | 2. (0.33, 13.) | - | - | - | - |
