# Supplementary material for: ADCY3: the pivotal gene in classical ketogenic diet for the treatment of epilepsy
Source: Front Cell Neurosci. 2024 May 22;18:1305867. doi: 10.3389/fncel.2024.1305867 (PMC11150708; doi:10.3389/fncel.2024.1305867)
Supplement: Supplementary file 10 [file Table_6.DOCX]

**Table S6. characteristics of the patients**

| Group | amount of examples | male | female | Average age (years) | Average duration of illness (months) |
| --- | --- | --- | --- | --- | --- |
| CAU | 30 | 13 | 17 | 14.53±2.32 | 12.23±2.71 |
| KD | 30 | 12 | 18 | 9.98±1.71 | 11.93±3.66 |
| p |  | 1.00 | | 0.143 | 0.719 |
